# Motivational and Valence‐Related Modulation of Sleep/Wake Behavior are Mediated by Midbrain Dopamine and Uncoupled from the Homeostatic and Circadian Processes

**DOI:** 10.1002/advs.202200640

**Published:** 2022-07-06

**Authors:** Karim Fifel, Amina El Farissi, Yoan Cherasse, Masashi Yanagisawa

**Affiliations:** ^1^ International Institute for Integrative Sleep Medicine (WPI‐IIIS) University of Tsukuba Tsukuba Ibaraki 305‐8577 Japan

**Keywords:** behavioral valence, dopamine, motivation, sleep regulation

## Abstract

Motivation and its hedonic valence are powerful modulators of sleep/wake behavior, yet its underlying mechanism is still poorly understood. Given the well‐established role of midbrain dopamine (mDA) neurons in encoding motivation and emotional valence, here, neuronal mechanisms mediating sleep/wake regulation are systematically investigated by DA neurotransmission. It is discovered that mDA mediates the strong modulation of sleep/wake states by motivational valence. Surprisingly, this modulation can be uncoupled from the classically employed measures of circadian and homeostatic processes of sleep regulation. These results establish the experimental foundation for an additional new factor of sleep regulation. Furthermore, an electroencephalographic marker during wakefulness at the theta range is identified that can be used to reliably track valence‐related modulation of sleep. Taken together, this study identifies mDA signaling as an important neural substrate mediating sleep modulation by motivational valence.

## Graphical abstract

It is demonstrated that motivational valence is a powerful modulator of the quality and quantity of sleep/wake behavior. This modulation of sleep/wake states by behavioral valence is mediated by midbrain dopamine neurons and is uncoupled from the homeostatic and circadian processes of sleep regulation.

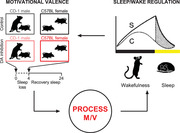

## 1 Introduction

Contextual salience as well as the extent of motivation it drives are powerful modulators of sleep/wake behavior.^[^ ^1^, ^2^, ^3^, ^4^, ^5^, ^6^ ^]^ Recent wild‐ and lab‐based studies have revealed an intriguing flexibility in the daily amount of sleep across several species.^[^ ^7^ ^]^ During postpartum period, dolphin and killer whale mothers and their calves show an almost total lack of sleep for several weeks.^[^ ^2^ ^]^ When in water, fur seals show no or greatly reduced rapid eye movement (REM) sleep for several consecutive days.^[^ ^5^ ^]^ Similarly, in several birds, total time spent sleeping can be greatly reduced during migration.^[^ ^1^, ^3^, ^4^, ^8^, ^9^ ^]^ Recent studies in fruit flies and bumblebee workers have revealed comparable temporary flexibility in total amount of sleep during, respectively, mating^[^ ^10^, ^11^, ^12^ ^]^ and caring of offspring.^[^ ^6^ ^]^ Two remarkable features characterize all these sleep adaptations; first, during the extended wake periods, learning abilities as well as neurobehavioral performances are intact or even higher. Second, no recovery sleep rebound is observed following these exceptionally long periods of sleep loss. The mechanisms behind these sleep/wake behavior adaptations are still unknown. Interestingly, these examples challenge also the predominant conceptual two process model of sleep regulation according to which the timing and intensity of sleep are governed, respectively, by circadian and homeostatic processes.^[^ ^13^, ^14^, ^15^ ^]^ More specifically, this influential model stipulates that homeostatic process is responsible for the accumulation of sleep pressure during wakefulness and its dissipation during subsequent sleep. The duration and intensity of sleep should therefore always correlate with the duration of prior wake episodes.^[^ ^13^, ^14^, ^15^ ^]^ Given the high predictive value of the two‐process model in diverse experimental conditions,^[^ ^15^ ^]^ its discrepancy with the above‐mentioned examples remains enigmatic. A potential clue toward understanding the neural mechanisms behind this conundrum relies on the third common denominator in all organisms undergoing adaptive sleeplessness, namely, motivational drive. Typically, all known examples of adaptive sleep loss occur in animals/insects during periods of heightened motivational state and of high intrinsic value (i.e., migration, sexual reproduction, caring of offspring).

Mechanistically, dopamine (DA) neurotransmission is a major regulator and/or modulator of motivational and value‐related neural processes.^[^ ^16^, ^17^, ^18^, ^19^, ^20^, ^21^ ^]^ Regarding sleep/wake regulation, even since its discovery in the 50's, DA is known as a powerful wake‐promoting neurotransmitter.^[^ ^22^, ^23^ ^]^ Several lesional and pharmacological studies in wild‐type and genetically modified animal models have confirmed the role of DA in arousal regulation.^[^ ^24^, ^25^ ^]^ Additionally, studies in humans with individual differences in DA function as a result of a polymorphism on the DA transporter (DAT) have implicated DA in the modulation of electroencephalographic (EEG) markers of the homeostatic regulation of sleep (i.e., slow‐wave activity (SWA)).^[^ ^26^ ^]^ Recent advances in opto‐ and chemogenetic neuronal manipulations have substantiated the role of DA in the maintenance of awake state and its modulation by salient environments.^[^ ^27^, ^28^, ^29^, ^30^, ^31^ ^]^ Collectively, these studies firmly established DA as a powerful regulator of arousal. However, the mechanisms by which this is achieved are still unknown. Basically, all previous studies have either examined the impact of DA manipulation on sleep/wake states^[^ ^27^, ^28^, ^29^, ^30^, ^31^, ^32^, ^33^ ^]^ or unraveled the downstream neuronal circuitry mediating these effects.^[^ ^34^, ^35^ ^]^ Whether DA modulates sleep/wake states by its interaction with the homeostatic and/or the circadian processes of sleep regulation is not fully understood. Additionally, the potential contribution of the acute masking effect of light and darkness^[^ ^36^, ^37^, ^38^ ^]^ to the sleep/wake alterations precipitated by DA modulation is unknown. Given the role of DA in encoding behavioral valence,^[^ ^17^ ^]^ a third important, but still unexplored, question is the role of valence in shaping sleep/wake behavior and its modulation by DA.

To address these questions, we combined chronic monitoring of sleep/wake states via EEG and electromyographic (EMG) recordings and selective and reversible chemogenetic approach to probe the mechanisms underlying sleep/wake alteration induced by silencing midbrain DA neurons in mice. We found that chronic inhibition of mDA neurons increased total sleep over the 24 h day. Interestingly, the magnitude of this increase was dependent on the contextual salience of the animals. Surprisingly, these alterations were not associated with any dysfunction in the homeostatic process nor in the fundamental properties of the circadian process of sleep regulation. Furthermore, the acute influence of light and dark on sleep could not account for the sleep alterations induced by DA silencing. Consistent with the motivational role of mDA neurons,^[^ ^18^ ^]^ and by manipulating sleep pressure in animals, we found that DA silencing decreased the threshold of inducing sleep in highly salient environments. Finally, we uncovered a new role of both DA and behavioral valence in mediating a significant modulation of sleep amount independently of sleep homoeostatic pressure. Collectively, our results provide strong evidence implicating DA‐mediated motivation and contextual valence as powerful factors in modulating sleep/wake behavior which were uncoupled from the classical homoeostatic and circadian processes of sleep regulation.

## 2 Results

### 2.1 Silencing Midbrain DA Neurons Induces Context‐Dependent Modulation of Total Amount of Sleep

**Figure 1:**
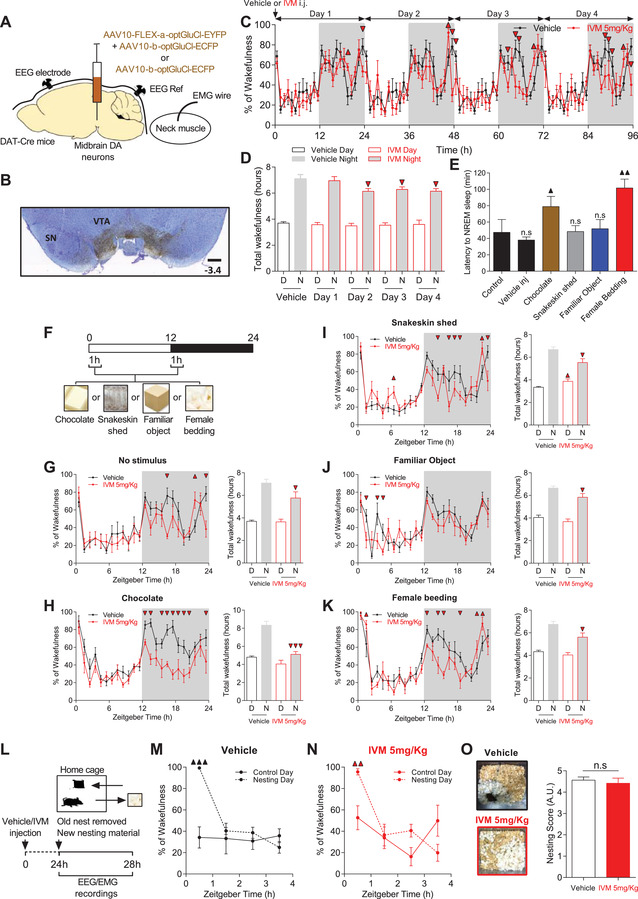
Silencing mDA neurons induces context‐dependent modulation of sleep/wake cycle. A) DAT‐Cre mice were injected with AAV10‐FLEX‐*α*‐optGluCl‐EYFP and AAV10‐*β*‐optGluCl‐ECFP or AAV10‐*β*‐optGluCl‐ECFP alone and implanted with EEG and EMG electrode to record sleep/wake states. B) Typical example of anti‐GFP staining revealing the extent of virus infection in the midbrain (Bregma = −3.4 mm; scale bar = 250 µm). Background staining is Cresyl Violet. C) Percentage of time spent in wakefulness before (vehicle) and over 4 days after IVM inj. (5 mg kg^−1^) in VTA^GluCl*αβ*^ mice. Vehicle day is quadri‐plotted for comparison (*n* = 6, two‐way RM ANOVA revealed days × time interaction, *F*_4,23_ = 1.537, *p* = 0.002; Bonferroni post hoc analysis, *p* < 0.05). D) Total time in wake during 12 h day (D) and 12 h night (N) before (vehicle) and over 4 days after 5 mg kg^−1^ IVM treatment (*n* = 6, two‐way RM ANOVA revealed no difference during the day but a significant difference during the night, *F*_1.4_ = 2.53, *p* = 0.04; Bonferroni post hoc analysis, *p* < 0.05). E) In control mice, latency to NREM sleep was significantly increased after the introduction of chocolate or female bedding (*n* = 8, one‐way RM ANOVA, *F*_5,40_ = 4.621, *p* = 0.002; Bonferroni post hoc analysis, ▲*p* < 0.05, ▲▲*p* < 0.01). F) Vehicle (*n* = 8) and IVM‐treated (*n* = 6) VTA^GluCl*αβ*^ mice were exposed to different salient stimuli during the first hour of light and dark phases simultaneously of recording their sleep/wake vigilance states. G–K) Left: Percentage of time spent in wakefulness in vehicle‐ and IVM‐treated VTA^GluCl*αβ*^ mice during G) baseline day (no stimuli), H) exposure to chocolate, I) snakeskin shed, J) familiar object, and K) female bedding (two‐way RM ANOVA revealed treatment × time interaction, *F*_1.23_(no stimuli) = 1.91, *p* = 0.008; *F*_1,23_(chocolate) = 2.315, *p* < 0.001; *F*_1,23_(snakeskin shed) = 2.791, *p* < 0.001; *F*_1,23_(familiar object) = 1.528, *p* = 0.061; *F*_1,23_(female bedding) = 2.394, *p* < 0.001; Bonferroni post hoc analysis, *p* < 0.05). Right: Total time in wake during 12 h day (D) and 12 h night (N) in both vehicle and IVM‐treated VTA^GluCl*αβ*^ mice (two‐way RM ANOVA revealed a significant difference during the night for all (*F*_1,1_(no stimuli) = 3.881, *p* = 0.045; *F*_1,1_(chocolate) = 12.863, *p* = 0.001; *F*_1,1_(snakeskin shed) = 14.455, *p* < 0.001; *F*_1,1_(familiar object) = 0.791, *p* = 0.024; *F*_1,1_(female bedding) = 2.644, *p* = 0.011; Bonferroni post hoc analysis, ▼ *p* < 0.05, ▼▼ *p* < 0.01, ▼▼▼ *p* < 0.001). L) Diagram depicting nest building behavior experiment. M,N) Percentage of time spent awake during the 4 h following the introduction of new nesting material in M) vehicle and N) IVM‐treated VTA^GluCl*αβ*^ mice (*n* = 6 per group, two‐way RM ANOVA revealed condition × time interaction for both groups, *F*_1,3_(vehicle) = 11.196, *p* < 0.001; *F*_1,3_ (IVM) = 4.84, *p* = 0.006, Bonferroni post hoc analysis, ▲▲▲ *p* < 0.0001, ▲▲ *p* = 0.003). O) Left: Representative pictures of vehicle (top) and IVM‐treated VTA^GluCl*αβ*^ (bottom) mouse cages at the end of 4 h EEG/EMG recordings. Right: Nesting score represents the amount of nesting material used and final shape of the nest during 1 h test period (1, poor; 5, good) (Wilcoxon matched‐pairs signed rank test). Data represent mean ± SEM.

To selectively silence DA neurons, we used GluCl (glutamate‐gated chloride)/IVM system for which targeting specificity as well as in vivo pharmacokinetics and pharmacodynamics was previously established.^[^ ^39^, ^44^ ^]^ This chemogenetic system involves a C‐elegans glutamate‐ and ivermectine (IVM)‐gated chloride‐channel that was mutagenetically modified to abolish sensitivity to glutamate while retaining sensitivity to IVM.^[^ ^39^ ^]^ The concept of this system is similar to other engineered receptors for neuronal silencing (i.e., designer receptors exclusively activated by designer drugs),^[^ ^45^ ^]^ but unlike others, allows for chronic silencing for several days after administration of IVM.^[^ ^39^, ^44^ ^]^ In the context of our study, this long window of IVM action will allow us to investigate the mechanisms of sleep alterations induced by silencing DA neurons without inherent stress and repetitive interference with animals’ behavior associated with other chemogenetic systems.^[^ ^45^ ^]^ The altered heteromeric GluCl channel is made up of two subunits (*α* and *β*). In order to selectively target DA neurons, we stereotaxically and bilaterally injected a mixture of two adeno‐associated virus (AAV) carrying a floxed *α*‐subunit or *β*‐subunit of the IVM‐gated GluCl channel into the ventral tegmental area (VTA) of DAT‐Cre mice (henceforth referred to as VTA^GluCl*αβ*^, **Figure** **1**A,B). Sham controls were injected only with the AAV carrying the *β*‐subunit (henceforth referred to as VTA^GluCl*β*^, Figure 1A). Although when administered i.p. at veterinary doses, IVM does not affect mouse behavior, at high concentrations, it can precipitate adverse effects on locomotor behavior in wild‐type mice.^[^ ^39^ ^]^ Our first experiments consisted therefore of determining the safe dose of IVM that is not associated with adverse side‐effects on sleep/wake behavior. To this end, after a 24 h baseline EEG/EMG recording, we treated sham‐control mice (Figure S1A, Supporting Information) with either 5 or 10 mg kg^−1^ of IVM and followed the impact of this treatment on subsequent 48 h sleep/wake behavior (Figure S1B, Supporting Information). 5 mg kg^−1^ did not affect sleep/wake behavior (Figure S1C–H, Supporting Information) while 10 mg kg^−1^ of IVM induced significant increase and decrease of, respectively, wake (Figure S1I, Supporting Information) and both REM (Figure S1K, Supporting Information) and nonrapid eye movement (NREM) sleep (Figure S1M, Supporting Information). As was shown before for locomotor behavior,^[^ ^39^ ^]^ 2.5 mg kg^−1^ of IVM was not effective in altering sleep/wake cycle even in DAT‐Cre mice injected with *α* and *β* subunits of the GluCl channel (VTA^GluCl*αβ*^, Figure S2A–G, Supporting Information). For our subsequent experiments, we therefore opted for 5 mg kg^−1^ of IVM to effectively silence midbrain DA neurons without inducing confounding side effects on sleep/wake behavior. Using double TH/c‐fos immunostaining in the midbrain of vehicle‐ and IVM‐treated VTA^GluCl*αβ*^, we confirmed the efficiency of the GluCl/IVM system to chronically silence DA neurons (Figure S3, Supporting Information).

VTA^GluCl*αβ*^ and VTA^GluCl*β*^ mice were habituated to handling for 1 week prior to i.p. injection of vehicle (propylene glycol) or IVM (5 mg kg^−1^) at the beginning of the light phase (ZT0) and EEG/EMG data were recorded for 4 consecutive days (Figure 1C,D). Compared to vehicle, IVM injection affected quantitatively sleep/wake cycle over the 4 following days (Figure 1C). Midbrain DA inhibition decreased the time spent in wakefulness during Day 2 to Day 4 after IVM injection (Figure 1D). Interestingly, this hypersomnia phenotype was restricted to the dark, active phases with no significant changes during the light, sleep phases (Figure 1D). Specifically, IVM injection into VTA^GluCl*αβ*^ mice increased the number of elongated NREM episodes (Figure S4A, Supporting Information) and overall NREM sleep duration (Figure S5C, Supporting Information) without affecting the number of transitions between different vigilance states (Figure S5A, Supporting Information) and the number of episodes of each state (Figure S5B, Supporting Information). This suggest that IVM‐treated VTA^GluCl*αβ*^ mice enter sleep as often as controls during the dark phase but elongated the duration of these sleep states. These observations corroborate recent studies demonstrating that chemogenetic or optogenetic inhibition of mDA neurons promotes sleep.^[^ ^27^, ^30^, ^31^, ^46^ ^]^

As a neuromodulator, DA allows for flexible, context‐dependent modulation of behavior.^[^ ^47^, ^48^ ^]^ We therefore asked whether silencing DA neurons induces a context‐dependent modulation of sleep/wake behavior. To do this, we examined the impact of DA inhibition on 24 h sleep/wake cycle in different motivational environments (Figure 1E–K). The contextual salience of the animals was modulated by introducing different salient stimuli into the cage of the animals during 1 h both in the beginning of light and dark phases of the light/dark (LD) cycle (i.e., chocolate, snakeskin shed, familiar rectangular object, and fresh female bedding, Figure 1E). To estimate the relative salience of the different stimuli, we measured in vehicle‐treated VTA^GluCl*αβ*^ mice the latency to NREM sleep after their introduction for 1 h (ZT00‐01) into animal's cage in the beginning of the sleep phase. We found that both chocolate and female bedding significantly increased sleep latency while snakeskin shedding, familiar object, and an i.p. vehicle injection did not change sleep latency relative to control (Figure 1E). These results show that these salient stimuli induce different motivational levels in mice. Introducing these salient stimuli to VTA^GluCl*αβ*^ mice after IVM (5 mg kg^−1^) administration revealed a differential and persistent alterations in sleep/wake behavior relative to vehicle‐treated VTA^GluCl*αβ*^ (Figure 1F–K). Interestingly, these alterations were mainly restricted to the dark phase of the LD cycle. The most dramatic deficits after DA inhibition were seen following the introduction of chocolate (Figure 1H) and female bedding (Figure 1K). While vehicle‐treated mice showed robust increases in wakefulness even after the removal of salient stimuli from the cages, such responses were absent after IVM administration leading to a significant decrease in total wakefulness over the dark phase (Figure 1H,K). Inhibiting mDA neurons also prevented the arousal response when an aversive stimulus (i.e., snakeskin shed) was introduced to animals as evidenced by a significant decrease in the percentage of wakefulness during the dark phase (Figure 1I). Unlike all the other salient stimuli, snakeskin shedding slightly but significantly increased total wakefulness states during the 7th h of the light phase of the LD cycle (Figure 1I). Finally, exposing IVM‐treated VTA^GluCl*αβ*^ mice to a familiar object induced comparable overall sleep/wake behavior phenotype (Figure 1J) as in animals not exposed to any salient stimuli (Figure 1G). Collectively, these findings demonstrate that the extent of sleep/wake alterations induced by mDA inhibition is modulated by the motivational context of the animals.

Lastly, we examined the impact of mDA inhibition on nest‐building behavior. A previous study implicated VTA DA neurons in the modulation of sleep‐related nesting behavior.^[^ ^27^ ^]^ 24 h after vehicle or IVM administration to VTA^GluCl*αβ*^ mice, new nesting material was introduced to animal's home cage at the beginning of the light phase when nesting behavior normally takes place and EEG/EMG signals were recorded for 4 h (Figure 1L). Both vehicle‐ and IVM‐treated VTA^GluCl*αβ*^ mice stayed awake during the first hour following the introduction of new nesting material leading to a significant increase in the percentage of wakefulness relative to a control day without the introduction of new nesting material (Figure 1M,N). During this hour, the animals were actively engaged in nest‐building without a significant difference between vehicle‐ and IVM‐treated mice as evidenced by the high scores achieved by both groups (Figure 1O). These results indicate that chronic midbrain DA inhibition does not affect sleep‐related nest building behavior in mice. Taken together, our results demonstrate that midbrain DA neuron inhibition promotes sleep and that the extent of this promotion is modulated by contextual salience.

### 2.2 Sleep Pressure and its Homeostatic Regulation is Not Affected by mDA Neurons Inhibition

**Figure 2:**
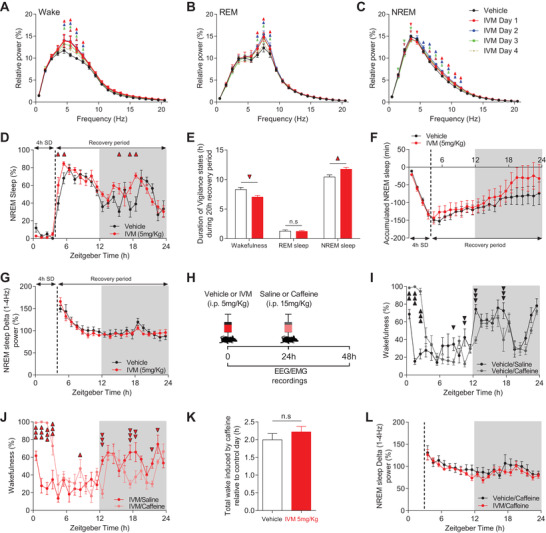
Chronic silencing of mDA neurons does not impact sleep pressure and its homeostatic regulation. A–C) Power spectral density analyses of A) wake, B) REM, and C) NREM sleep during 24 h vehicle day and over 4 days following IVM (5 mg kg^−1^) treatment of VTA^GluCl*αβ*^ mice (*n* = 6, two‐way RM ANOVA revealed days × frequency interaction. *F*_4,20_(wake) = 2.982, *p* < 0.001; *F*_4,20_(REM) = 2.601, *p* < 0.001; *F*_4,20_(NREM) = 4.046, *p* < 0.001; Bonferroni post hoc analysis, *p* < 0.05. D) Percentage of time spent in NREM sleep during 4 h SD day in vehicle (*n* = 8) and IVM‐treated (*n* = 6) VTA^GluCl*αβ*^ mice (two‐way RM ANOVA revealed a significant Groups × time interaction. *F*_1,23_ = 2.178, *p* = 0.002; Bonferroni post hoc analysis, *p* < 0.05). E) Total time spent in wake, REM, and NREM sleep during recovery period in vehicle and IVM‐treated VTA^GluCl*αβ*^ mice (one‐way RM ANOVA revealed significant decrease and increase of wake and NREM sleep, respectively, after DA neurons inhibition, *F*_1,12_(wake) = 7.314, *p* = 0.019; *F*_1,12_(REM sleep) = 0.024, *p* = 0.878; *F*_1,12_(NREM sleep) = 6.599, *p* = 0.025). F) Cumulative recovery NREM sleep during SD day (two‐way RM ANOVA revealed no significant Groups × time interaction. *F*_1,23_ = 0.492, *p* = 0.977). G) Evolution of NREM sleep delta power after 4 h SD expressed as percentage of total NREM sleep delta power (1–4 Hz) during 20 h recovery period (two‐way RM ANOVA revealed no Groups × time interaction *F*_1,23_ = 0.636, *p* = 0.902). H) Diagram depicting caffeine treatment. I,J) In both I) vehicle and J) IVM‐treated VTA^GluCl*αβ*^ mice, caffeine (15 mg kg^−1^) significantly promoted wakefulness (two‐way RM ANOVA revealed significant Groups × time interaction. *F*_1,23_(vehicle) = 8.68, *p* < 0.001; *F*_1,23_(IVM) = 8.424, *p* < 0.001; Bonferroni post hoc analysis, 1 triangle *p* < 0.05; 2 triangles, *p* < 0.01; 3 triangles *p* < 0.001). K) No difference in total wake induced during 4 h following caffeine injection between vehicle and IVM‐treated VTA^GluCl*αβ*^ mice (unpaired *t* test). L) Evolution of NREM sleep delta power after caffeine treatment (two‐way RM ANOVA revealed no significant difference between the groups. *F*_1257_ = 0.0002, *p* = 0.987). Data represent mean ± SEM.

By what mechanism does DA neurons silencing promotes NREM sleep? According to the classical model of sleep regulation, time spent in sleep is determined by a homeostatic sleep need which increases during wakefulness and dissipates during sleep.^[^ ^13^, ^14^ ^]^ The spectral power density of EEG delta waves (1–4 Hz) during NREM sleep is the best electrophysiological marker of sleep depth.^[^ ^13^, ^14^, ^15^ ^]^ We analyzed the spectral composition of the EEG separately for wake, REM, and NREM sleep before and after IVM administration (**Figure** **2**A–C). Surprisingly, after IVM injection, VTA^GluCl*αβ*^ mice exhibited a slight but significant reduction in the density of SWA during NREM sleep (Figure 2C) suggesting that increased NREM sleep after mDA inhibition is not driven by increased sleep pressure. To examine whether the modulation of sleep time by contextual salience in IVM‐treated VTA^GluCl*αβ*^ mice is mediated also independently of sleep pressure, we examined the 24 h dynamic and overall power density of delta waves during NREM sleep of the different salient contexts (Figure S6A–K, Supporting Information). Overall, and despite the significant differences in total 24 h NREM sleep (Figure 1F–K), we did not find corresponding changes in delta power during NREM sleep between these different environments after mDA inhibition (Figure S6B,D,F,H,J, Supporting Information) and the 24 h total power densities of delta waves during NREM sleep were identical to controls in all salient conditions (Figure S6C,E,G,I,K, Supporting Information). These results demonstrate that the contextual modulation of the amount of NREM sleep after the inhibition of mDA neurons is uncoupled from changes in sleep pressure.

To assess the homeostatic regulation of sleep after mDA neurons inhibition, we challenged vehicle‐ and IVM‐treated VTA^GluCl*αβ*^ mice with 4 h sleep deprivation (SD) starting from the onset of the light phase. Both groups responded to SD by sleeping more during the recovery period relative to baseline. In few occasions, IVM‐treated VTA^GluCl*αβ*^ mice showed significant increases in NREM sleep (Figure 2D) leading to a slight increase in total NREM sleep during the 20 h recovery period (Figure 2E). The percentage of REM sleep was not affected after SD in IVM‐ versus vehicle‐treated VTA^GluCl*αβ*^ mice (Figure 2E). The slight increase in NREM sleep after SD in IVM‐treated VTA^GluCl*αβ*^ mice could reflect either an altered homeostatic response to SD or the intrinsic hypersomnia induced by DA neuron inhibition. To distinguish between these two possibilities, we calculated the accumulated NREM sleep over the SD day in vehicle‐ and IVM‐treated VTA^GluCl*αβ*^ mice relative to their respective NREM sleep during baseline (Figure 2F). Both groups displayed similar dynamic of NREM sleep loss and recovery during, respectively, SD and recovery periods (Figure 2F). Additionally, NREM sleep delta power during recovery period was indistinguishable between vehicle‐ and IVM‐treated VTA^GluCl*αβ*^ mice (Figure 2G). These results demonstrate that DA neurons inhibition does not alter the homeostatic response to sleep deprivation.

To corroborate this conclusion, we next examined whether IVM‐treated VTA^GluCl*αβ*^ mice show an unaltered wake‐promoting response to caffeine (Figure 2H–L). The wake‐promoting effect of caffeine is mediated by antagonizing the action of adenosine which is considered one of the main mediators of homeostatic regulation of sleep in the brain.^[^ ^49^ ^]^ 24 h after vehicle or IVM (5 mg kg^−1^) i.p. injection to VTA^GluCl*αβ*^ mice, animals were administered either 15 mg kg^−1^ of caffeine or saline at the beginning of the light phase (Figure 2H). As expected, caffeine promoted comparable wake response in IVM‐ versus vehicle‐treated VTA^GluCl*αβ*^ mice (Figure 2I,J). The total wake induced by caffeine during the first 4 h of light phase was not different between IVM‐ and vehicle‐treated VTA^GluCl*αβ*^ mice (Figure 2K). Furthermore, and similar to SD response, the dynamic of NREM sleep delta power after caffeine injection was not affected after IVM treatment (Figure 2L). These results confirm the unaltered homeostatic regulation of sleep following sleep loss induced by caffeine after silencing DA neurons. Taken together, our results show that the hypersomnia induced by inhibition of DA neurons is not mediated by increased sleep pressure nor by an altered homeostatic regulation of sleep/wake behavior.

### 2.3 The Fundamental Properties of the Circadian Clock Remain Intact after mDA Neurons Inhibition

**Figure 3:**
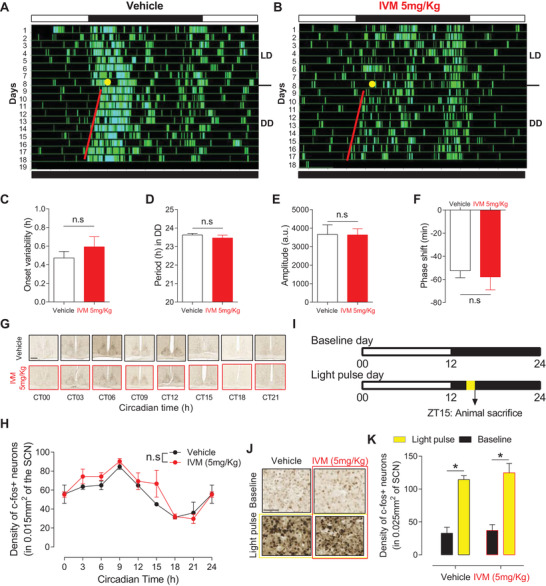
Chronic silencing of mDA neurons does not impair neither the endogenous function of the circadian clock nor its response to light. A,B) Representative locomotor activity actograms from A) vehicle and B) IVM‐treated VTA^GluCl*αβ*^ mice. Mice were initially entrained to 12 h/12 h LD cycle (LD) then released into continuous darkness (DD). Before releasing to DD, mice were exposed to 15 min light pulse at ZT14 (yellow circles). C) The variability around activity onset was not significantly affected following IVM treatment (*n* = 6 per group, paired *t* test, *t*_5_ = −1.379, *p* = 0.226). D) The endogenous period of rest/activity cycle was not different between vehicle and IVM‐treated mice (paired *t* test, *t*_5_ = 2.177, *p* = 0.081). E) The amplitude of rest/activity rhythm was not different as well (paired *t* test, *t*_5_ = 0.0689, *p* = 0.948). F) The extent of the phase shift induced by a 15 min light pulse was not different between vehicle and IVM‐treated VTA^GluCl*αβ*^ mice (paired *t* test, *t*_5_ = 0.326, *p* = 0.758). G,H) Immunohistochemical analysis shows that the circadian rhythm of c‐fos expression is not affected in the SCN of IVM‐treated VTA^GluCl*αβ*^ mice (*n* = 3 per time point, two‐way ANOVA, *F*_1,7_ = 0.860, *p* = 0.548). Scale bar in (G) = 250 µm. I) Diagram depicting the 1 h light exposure essay. J,K) Immunohistochemical analysis shows that the extent of c‐fos expression at baseline and after 1 h light exposure (at ZT14 to 15) was not affected following IVM treatment of VTA^GluCl*αβ*^ mice (two‐way ANOVA, *F*_1,1_ = 0.0719, *p* = 0.793; *p < 0.001). Scale bar in (J) = 50 µm. Data represent mean ± SEM.

The second process of sleep regulation involves the circadian system.^[^ ^13^, ^14^ ^]^ The endogenous clock in the suprachiasmatic nucleus (SCN) is primarily involved in the timing of wake and sleep behaviors within the geophysical day.^[^ ^50^ ^]^ However, in addition to these qualitative aspects of sleep/wake regulation, recent evidence has implicated the molecular clock in shaping quantitative aspects of sleep/wake cycle as well.^[^ ^15^, ^51^ ^]^ We therefore sought to examine whether mDA neurons inhibition affects the fundamental properties of the circadian clock. We monitored daily changes in rest/activity rhythm in vehicle‐ and IVM‐treated VTA^GluCl*αβ*^ mice (**Figure** **3**). Animals were maintained in 12 h/12 h LD cycles and then released into constant darkness. Although IVM‐treated VTA^GluCl*αβ*^ mice displayed a slight nonsignificant increase in their activity onset variability (Figure 3C), they showed normal entrainment to LD cycle and a normal clock‐controlled free‐running rest/activity rhythm in constant darkness (Figure 3A,B). Analyses of the free‐running behavior of mice in constant DD revealed no difference between vehicle‐ and IVM‐treated VTA^GluCl*αβ*^ mice in both the period (Figure 3D) and the amplitude of the rest/activity rhythm (Figure 3E). Within the SCN, the circadian pattern of c‐fos expression was unchanged between IVM‐treated and vehicle‐treated VTA^GluCl*αβ*^ mice (Figure 3G,H). These results demonstrate that the fundamental properties of the master clock after inhibition of mDA neurons are not impaired. To confirm the normal function of the circadian clock, we evaluated the photic response of the rest/activity rhythm in terms of the extent of the phase shift induced by a 15 min light pulse given at Zeitgeber time (ZT) 14 (Figure 3A,B). We found no significant difference in the induced phase delays between vehicle‐ and IVM‐treated VTA^GluCl*αβ*^ mice (Figure 3F). Similarly, IVM treatment did not affect the extent of light‐induced expression of c‐fos expression in the SCN (Figure 3J,K). Collectively, these findings confirm the functional integrity of the circadian system which implies therefore that sleep/wake alterations precipitated by chronic inhibition of mDA neurons are not mediated by a dysfunctional circadian process of sleep regulation.

### 2.4 The Acute Modulation of Sleep/Wake Behavior by Light and Dark Remains Broadly Intact after mDA Inhibition

**Figure 4:**
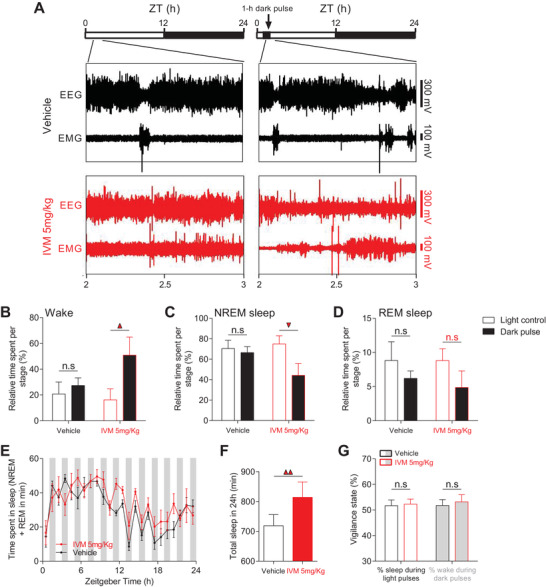
Acute masking effects of light and dark are broadly intact after mDA neurons inhibition. A) Representative EEG and EMG traces of vehicle (upper) and IVM‐treated (lower) VTA^GluCl*αβ*^ mice at ZT2‐3 without (left) and with (right) dark pulse exposure. Light schedules are depicted by black and white bars above the panels. B–D) Quantification of dark pulse effects on different sleep/wake states at ZT2‐3 (vehicle, *n* = 8; IVM, *n* = 6; *F*_1,1_(wake) = 2.175, *p* = 0.153; *F*_1,1_(NREM) = 2.525, *p* = 0.125; *F*_1,1_(REM) = 0.09, *p* = 0.762; Bonferroni post hoc analysis, triangle, *p* < 0.05). E) Time spent in sleep (NREM + REM sleep) during 1 h light/1 h dark protocol in vehicle and IVM‐treated VTA^GluCl*αβ*^ mice (*n* = 7 per group, two‐way RM ANOVA revealed no Groups × time interaction. *F*_1,23_ = 1.078, *p* = 0.378). F) IVM‐treated mice spent significantly more time asleep over 24 h of the 1 h/1 h LD protocol relative to vehicle‐treated mice (*n* = 7 per group, paired *t* test, *t*_6_ = −3.887, *p* = 0.008). G) The relative distribution of sleep and wake during light and dark phase was not different between vehicle and IVM‐treated VTA^GluCl*αβ*^ mice (*n* = 7 per group, paired *t* test, *t*_6_(%sleep) = −0.161, *p* = 0.877; *t*_6_(%wake) = −0.308, *p* = 0.768). Data represent mean ± SEM.

In addition of its photoentrainment properties,^[^ ^53^ ^]^ light and dark exert an acute and direct impact on the sleep/wake cycle known as masking.^[^ ^36^, ^37^, ^38^ ^]^ In nocturnal rodents, light strongly induces sleep whereas darkness is wake promoting.^[^ ^54^ ^]^ Therefore, a potential mechanism behind the hypersomnia induced in IVM‐treated VTA^GluCl*αβ*^ mice during the dark phase could be an impaired wake promotion by darkness. To explore this possibility, we examined wake induction in vehicle‐ and IVM‐treated VTA^GluCl*αβ*^ mice by exposing them to a 1 h dark pulse starting 2 h after “light‐on” (ZT02, **Figure** **4**A). The distribution of different sleep/wake states throughout the 60 min dark exposure was not affected in vehicle‐treated VTA^GluCl*αβ*^ mice (Figure 4B–D). Relative to vehicle, IVM‐treated VTA^GluCl*αβ*^ mice showed a significant increase and decrease in, respectively, wake (Figure 4B) and NREM sleep (Figure 4C) percentage after dark pulse exposure with no impact on REM sleep (Figure 4D). We then examined the light‐induction of sleep before and after DA silencing (Figure S7, Supporting Information). To this end, we exposed vehicle‐ and IVM‐treated VTA^GluCl*αβ*^ mice to a 1 h light pulse 2 h after “light‐off” (ZT14, Figure S7A, Supporting Information). As expected, in vehicle‐treated mice, light pulse of 300 lux increased the time spent in NREM sleep by ≈60% (Figure S7C, Supporting Information) whereas wake state was correspondingly decreased (Figure S7B, Supporting Information). No significant changes were seen for REM sleep (Figure S7D, Supporting Information). In IVM‐treated VTA^GluCl*αβ*^ mice, light‐mediated sleep induction was affected. No significant changes were observed for all vigilance states during light exposure relative to a corresponding ZT time without light exposure (Figure S7B–D, Supporting Information). Interestingly, the effect of mDA inhibition on the impact of masking on sleep/wake behavior during the examined time windows (respectively, ZT2‐3 and ZT14‐15) is the opposite of what we would expect if this mechanism is contributing to the sleep phenotype induced in IVM‐treated VTA^GluCl*αβ*^ mice. We therefore sought to probe in more detail the effects of light on sleep across the 24 h day in order to obtain a more complete account of the acute effect of light and dark on sleep/wake behavior following DA inhibition. To achieve this, we exposed vehicle‐ and IVM‐treated VTA^GluCl*αβ*^ mice to an ultradian 24 LD cycle (1 h/1 h LD). Mice are not able to photoentrain to this short cycle, hence light and dark pulses will fall within all phases of the circadian cycle.^[^ ^37^, ^54^ ^]^ In both vehicle‐ and IVM‐treated VTA^GluCl*αβ*^ mice, light pulses were found to repetitively induce sleep, however not consistently throughout the circadian cycle (Figure 4E). In addition, quantification of sleep throughout the ultradian cycles revealed an obvious circadian rhythm in both vehicle‐ and IVM‐treated VTA^GluCl*αβ*^ mice (Figure 4E). These results agree with studies using a similar protocol in wild‐type mice.^[^ ^37^ ^]^ Again, IVM‐treated VTA^GluCl*αβ*^ mice showed significant increase in total sleep over the 24 h day (Figure 4F). However, the relative distribution of sleep and wake states percentages throughout all light and dark pulses was not different between vehicle‐ and IVM‐treated VTA^GluCl*αβ*^ mice (Figure 4G). These results demonstrate that the acute modulation of sleep/wake cycle by light and dark pulses is broadly unaffected following mDA neurons inhibition. Therefore, the acute masking effect of alternating light/dark cycles cannot account for the hypersomnia induced by mDA neurons inhibition.

### 2.5 Inhibition of mDA Neurons Induces an Impaired Arousal Response to, and a Lower Threshold of Sleep Induction in Face of, Salient Stimuli

**Figure 5:**
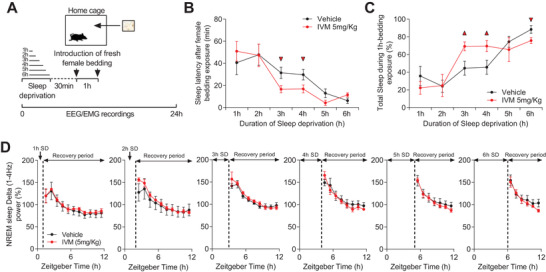
mDA neurons silencing impairs arousal response to salient stimuli. A) Diagram depicting the experimental protocol. B) Sleep latencies after 1 to 6 h SD and following exposure to fresh female bedding in vehicle‐ and IVM‐treated VTA^GluCl*αβ*^ mice (vehicle, *n* = 8; IVM, *n* = 6; unpaired *t* test, triangle, *p* < 0.05). C) Total time spent asleep during 1 h exposure to female bedding after 1 to 6 h SD (unpaired *t* test, triangle, *p* < 0.05). D) NREM sleep delta power after 1 to 6 h SD (two‐way RM ANOVA revealed no Groups × time interaction, *F*_1,10_(1hSD) = 0.023, *p* = 1; *F*_1,9_(2hSD) = 0.29, *p* = 0.976; *F*_1,8_(3hSD) = 0.486, *p* = 0.863; *F*_1,7_(4hSD) = 0.521, *p* = 0.816; *F*_1,6_(5hSD) = 0.099, *p* = 0.996; *F*_1,5_(6hSD) = 0.259, *p* = 0.934). Data represent mean ± SEM.

If the homeostatic (Figure 2) and circadian processes (Figure 3) of sleep regulation as well as the acute modulation of sleep/wake behavior by light (Figure 4) are all normal, what could account for the hypersomnia induced by chronic DA neurons inhibition? In addition of its wake promoting effect, recent studies have attributed to DA the role of encoding the value of work.^[^ ^16^, ^17^, ^18^, ^19^, ^20^, ^21^ ^]^ According to these studies, by encoding state and motivational value, DA increases motivational vigour as the animal approaches a reward target. Accordingly, DA is considered to encode instrumental wakefulness. We therefore hypothesize that the hypersomnia precipitated by mDA neurons inhibition results from an impaired DA‐mediated invigoration of instrumental wakefulness. To test this hypothesis, we subjected vehicle‐ and IVM‐treated VTA^GluCl*αβ*^ mice to different and increasing durations of sleep deprivations (1 to 6 h, starting from ZT0) in order to generate different psychological states with increasing sleep pressure. 30 min after the end of SD, we introduced fresh female bedding into animal's home cage and calculate sleep latency (**Figure** **5**A). The level of arousal is inversely linked to the likelihood of falling asleep and correlates positively with sleep latency. Additionally, the vigor of instrumental arousal is regulated independently of sleep need^[^ ^43^ ^]^ and reflects the sum activity of wake‐promoting neurons.^[^ ^55^ ^]^ An impaired DA‐mediated invigoration of instrumental wakefulness could be reflected therefore in impaired sleep latencies in face of salient stimuli. As expected, SD shortened the latencies to enter sleep in both vehicle‐ and IVM‐treated VTA^GluCl*αβ*^ mice, with shorter latencies in the more sleep‐deprived animals (Figure 5B). We found no significant difference in sleep latencies between the two groups after 1 and 2 h of SD (Figure 5B). However, starting from 3 h, IVM‐treated VTA^GluCl*αβ*^ mice had shorter latencies to fall asleep after introducing female bedding (Figure 5B). These latencies were ≈50% lower relative to the latencies in vehicle‐treated VTA^GluCl*αβ*^ mice (Figure 5B). After 5 and 6 h SD, both vehicle‐ and IVM‐treated VTA^GluCl*αβ*^ mice displayed very short sleep latencies with no significant differences between the two (Figure 5B), possibly owing to a ceiling effect.

Consistent with sleep latency data, the total amount of sleep (NREM + REM sleep) within the 1 h exposure to female bedding was significantly higher in IVM‐treated VTA^GluCl*αβ*^ mice following 3 and 4 h of SD with no difference after 1, 2, and 5 h of SD relative to vehicle‐treated VTA^GluCl*αβ*^ mice (Figure 5C). After 6 h SD, IVM‐treated mice showed a slight decrease in total sleep relative to vehicle‐treated mice. However, both groups showed high percentage (>60%) of sleep suggesting again a ceiling effect (Figure 5C). These results confirm our hypothesis that IVM‐treated VTA^GluCl*αβ*^ mice suffer from an impaired invigoration of instrumental wakefulness and display a lower threshold of sleep induction even within salient contexts.

Next, we examined whether these differential sleep responses between vehicle‐ and IVM‐treated VTA^GluCl*αβ*^ mice are mediated by differential sleep pressure between the two groups during the female bedding exposure test. To do this, we analyzed the dynamic of NREM sleep delta power in vehicle‐ and IVM‐treated VTA^GluCl*αβ*^ mice following SD experiments (Figure 5D). Consistent with our previous data (Figure 2 and Figure S6, Supporting Information), we found no significant difference in the dynamic of delta (1–4 Hz) power between the two groups (Figure 5D). This result demonstrates that vehicle‐ and IVM‐treated VTA^GluCl*αβ*^ mice had similar sleep depth after SD experiments, hence, an exacerbated sleep pressure could not account for the low threshold of sleep initiation in IVM‐treated VTA^GluCl*αβ*^ mice. Collectively, our data demonstrate that lowering DA tone decreases instrumental wakefulness and lowers the threshold of sleep induction without affecting the homeostatically regulated SWA.

### 2.6 Midbrain DA Neurons Mediate Valence‐Related Modulation of NREM Sleep Amount

**Figure 6:**
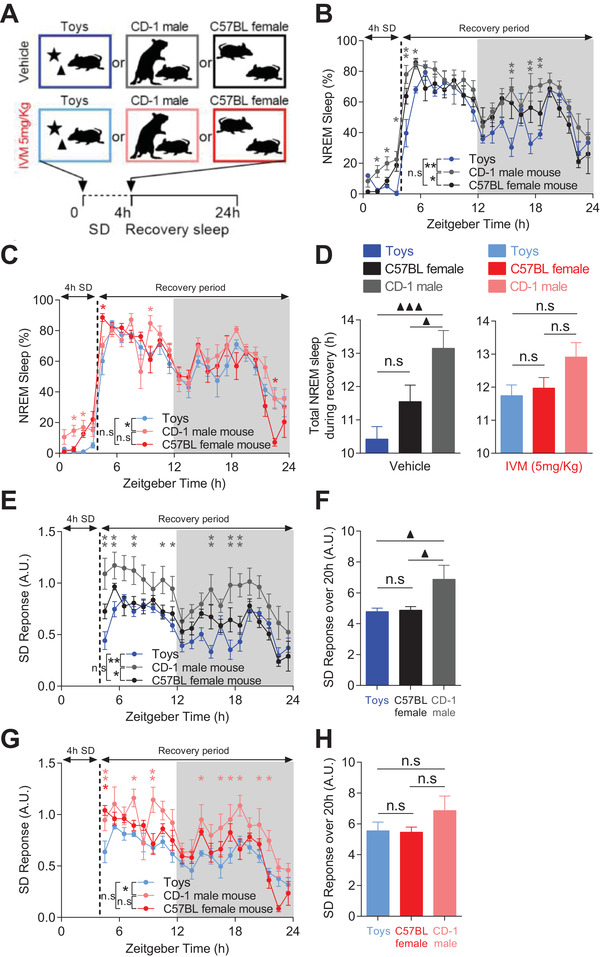
mDA neurons mediates valence‐related modulation of NREM sleep. A) Diagram depicting the experimental protocol. B,C) Percentage of time spent in NREM sleep in B) vehicle‐treated and C) IVM‐treated VTA^GluCl*αβ*^ mice during and after SD with novel toys, CD‐1 male or conspecific female interaction (*n* = 6 per group, two‐way RM ANOVA revealed significant Groups effect on NREM sleep during recovery for both vehicle‐ (*F*_2,23_ = 12.876, *p* = 0.002) and IVM‐treated mice (*F*_2,23_ = 5.760, *p =* 0.022), Bonferroni post hoc analysis, *p < 0.05, **p < 0.01). D) Total time spent in NREM sleep during recovery period in vehicle and IVM‐treated VTA^GluCl*αβ*^ mice after SD (one‐way RM ANOVA revealed significant difference in vehicle‐treated (*F*_2,10_ = 11.835, *p* = 0.002) but not in IVM‐treated mice (*F*_2,10_ = 3.476, *p* = 0.071), Bonferroni post hoc analysis, ▲▲▲ *p* < 0.001, ▲ *p* < 0.05). E) Hourly and F) overall normalized SD response calculated as [recovery sleep/(baseline sleep‐SD sleep)] in vehicle‐treated VTA^GluCl*αβ*^ mice (in (E) two‐way RM ANOVA, *F*_2,19_ = 7.988, *p* = 0.008, Bonferroni post hoc analysis,*p < 0.05, **p < 0.01; in (F) one‐way RM ANOVA, *F*_2,10_ = 4.247, *p* = 0.046, Bonferroni post hoc analysis, ▲ *p* < 0.05). G,H) Same as in (E,F) but for IVM‐treated VTA^GluCl*αβ*^ mice (in (G) two‐way RM ANOVA, *F*_2,19_ = 5.556, *p* = 0.024, Bonferroni post hoc analysis,*p < 0.05, **p < 0.01; in (H) one‐way RM ANOVA, *F*_2,10_ = 4.247, *p* = 0.43). Data represent mean ± SEM.

In addition of encoding motivational salience, DA neurons encode also motivational value.^[^ ^17^, ^56^ ^]^ Inspired by the remarkable motivation‐related adaptations of sleep amount recently described in lab conditions as well as in the wild,^[^ ^1^, ^2^, ^3^, ^4^, ^5^, ^6^, ^8^, ^9^, ^10^, ^11^, ^12^ ^]^ we sought to examine whether contrasting motivational values during instrumental wakefulness will differentially modulate subsequent sleep/wake behavior. Individually housed vehicle‐ and IVM‐treated VTA^GluCl*αβ*^ mice were subjected, in addition to the standard SD with novel objects, to two SD protocols with opposite valences for 4 h starting from lights on (**Figure** **6**A). The positively charged SD consisted of introducing a conspecific female intermittently into the animal's cage for 4 h while the negatively charged SD consisted of an acute social defeat induced by introducing an aggressive male CD‐1 mouse (see the Experimental Section for details). In vehicle‐treated VTA^GluCl*αβ*^ mice, the two SD protocols induced different sleep rebound phenotypes relative to SD with novel toys (Figure 6B). After SD, vehicle‐treated VTA^GluCl*αβ*^ mice that underwent social defeat with CD‐1 male spent significantly more time in NREM sleep at multiple occasions during recovery period relative to vehicle‐treated VTA^GluCl*αβ*^ mice that were sleep deprived with a conspecific female interaction (Figure 6B). This led to a significantly higher (by ≈2.5 h) cumulative NREM sleep time during recovery period in CD‐1‐exposed relative to toys‐exposed vehicle‐treated VTA^GluCl*αβ*^ mice (Figure 6D). Interestingly, CD‐1‐exposed vehicle‐treated VTA^GluCl*αβ*^ mice slept more during recovery period despite having lost less sleep during SD compared to both toys‐ and female‐exposed vehicle‐treated VTA^GluCl*αβ*^ mice (Figure 6B). Consistent with previous literature,^[^ ^57^ ^]^ the two SD protocols affected differently REM sleep rebound (Figure S8, Supporting Information). Relative to toys‐ and female‐exposed mice, exposure to CD‐1 male mice induced a powerful suppression of REM sleep after SD in vehicle‐treated VTA^GluCl*αβ*^ mice (Figure S8A,B, Supporting Information). These results demonstrate that the valence of the behavior during wakefulness is a powerful modulator of the quality and quantity of subsequent sleep.

Next, we wanted to examine the potential role of DA in mediating this differential modulation of sleep amount by behavioral valence. We therefore exposed IVM‐treated VTA^GluCl*αβ*^ mice to the same SD protocols (Figure 6A). Unlike vehicle‐treated VTA^GluCl*αβ*^ mice, IVM‐treated mice displayed a slight increase in NREM sleep rebound only during the 6th h following SD with CD‐1 male mice interaction relative to SD with novel toys (Figure 6C). Consequently, no significant difference was found in the cumulative NREM sleep time during recovery period between the 3 SD protocols (Figure 6D). IVM treatment of VTA^GluCl*αβ*^ mice did not affect the differential impact of behavioral valence on subsequent REM sleep amount (Figure S8C,D, Supporting Information). As a control experiment for potential side effects of IVM treatment, we treated wild‐type mice with IVM (i.p. 5 mg kg^−1^) and subjected them to the same contrasting SD protocols (Figure S9A, Supporting Information). IVM‐treatment did not affect valence‐related modulation of both NREM (Figure S9B,C, Supporting Information) and REM sleep (Figure S9D,E, Supporting Information) demonstrating that its impairment in IVM‐treated VTA^GluCl*αβ*^ mice (Figure 6) is not attributed to IVM treatment per se but rather to the successful inhibition of mDA neurons.

Challenging the classical model of homeostatic sleep regulation that considers time spent awake as the main determinant of sleep pressure,^[^ ^13^, ^14^ ^]^ in both vehicle‐ and IVM‐treated VTA^GluCl*αβ*^ mice, the NREM sleep rebound was stronger following SD with CD‐1 males despite having lost less NREM sleep during SD relative to toys‐ and conspecific female‐exposed mice (Figure 6B,C). To account for this, we normalized the percentage of NREM sleep during recovery period relative to the percentage of NREM sleep lost during SD (see Experimental Section). In vehicle‐treated VTA^GluCl*αβ*^ mice, the 3 SD protocols led to different SD responses with significantly higher responses in CD‐1 male relative to toys‐exposed mice throughout recovery period (Figure 6E). The total SD response during recovery period was therefore significantly higher in CD‐1 male compared to toys‐ and female‐exposed vehicle‐treated VTA^GluCl*αβ*^ mice (Figure 6F). In IVM‐treated VTA^GluCl*αβ*^ mice, this differential modulation of SD response by behavioral valence was significantly impaired (Figure 6G,H). Although CD‐1 male‐exposed IVM‐treated VTA^GluCl*αβ*^ mice still displayed significantly higher responses at few occasions during recovery period (Figure 6G), the cumulative SD response during recovery period was not statistically different between toys‐, CD‐1 male‐, and conspecific female‐exposed IVM‐treated VTA^GluCl*αβ*^ mice (Figure 6H). These results implicate mDA signaling in the mediation of sleep modulation by behavioral valence.

### 2.7 Valence Modulates Sleep Amount Independently of SWA Modulation

**Figure 7:**
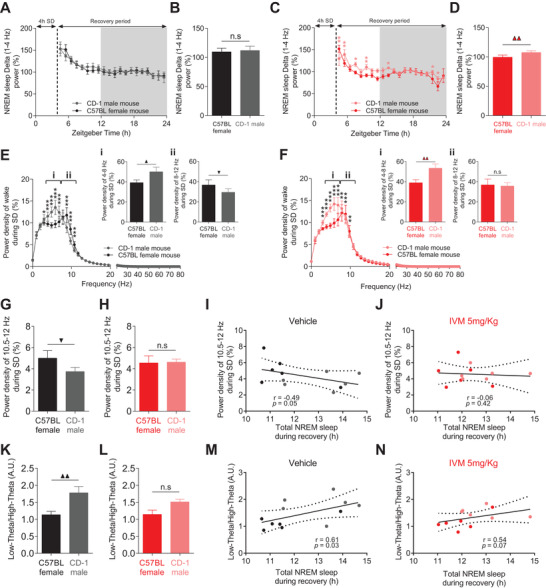
Valence‐related modulation of sleep correlates with [Low theta/High theta] index during wakefulness. A,B) Despite their different sleep rebound phenotype, no difference in the A) dynamic and B) overall NREM sleep delta power during recovery period between CD1 male‐exposed and conspecific female‐exposed vehicle‐treated VTA^GluCl*αβ*^ mice (*n* = 6, for (A) two‐way RM ANOVA revealed no significant Groups × time interaction. *F*_1,19_ = 0.521, *p* = 0.947. For (B) paired *t* test, *t*_5_ = −0.269, *p* = 0.802). C,D) Same as in (A, B) but for IVM‐treated VTA^GluCl*αβ*^ mice (*n* = 6, for (C) two‐way RM ANOVA revealed Groups × time interaction. *F*_1,19_ = 2.323, *p* = 0.004, Bonferroni post hoc analysis, *p < 0.05, **p < 0.01. For (D) paired *t* test, *t*_5_ = −5.489, *p* = 0.003). E) Power spectral density of EEG during SD with CD‐1 male and conspecific female in vehicle‐treated VTA^GluCl*αβ*^ mice (*n* = 6, two‐way RM ANOVA revealed a significant Groups × time interaction. *F*_1,81_ = 5.552, *p* < 0.001, Bonferroni post hoc analysis, *p < 0.05, ***p < 0.001). Relative to conspecific female‐exposed mice, CD‐1 male‐exposed vehicle‐treated VTA^GluCl*αβ*^ mice had significantly E‐i) higher and E‐ii) lower power densities of low theta (4–8 Hz) and high theta (8–12 Hz), respectively (paired *t* test, *t*_5_(low theta) = −2.589, *p* = 0.049, *t*_5_(high theta) = 3.462, *p* = 0.018). F) Same as (E) for IVM‐treated VTA^GluCl*αβ*^ mice (F) two‐way RM ANOVA. *F*_1,81_ = 6.505, *p* < 0.001. Bonferroni post hoc analysis, *p < 0.05, **p < 0.01, ***p < 0.001. F‐i,F‐ii) paired *t* test, *t*_5_(low theta) = −5.575, *p* = 0.003; *t*_5_(high theta) = 0.285, *p* = 0.787). G,H) Power density of EEG during SD at (10.5–12 Hz) significantly discriminated between CD‐1 male‐exposed and conspecific female‐exposed in G) vehicle‐treated VTA^GluCl*αβ*^ mice but not in H) IVM‐treated VTA^GluCl*αβ*^ mice (paired *t* test, *t*_5_(vehicle) = 2.917, *p* = 0.033, *t*_5_(IVM) = −0.104, *p* = 0.922). I,J) Correlations between power density of EEG during SD at (10.5–12 Hz) and total NREM sleep during recovery in I) vehicle‐ and J) IVM‐treated VTA^GluCl*αβ*^ mice (Spearman correlation). K,L) [Low theta/High theta] significantly discriminated between CD‐1 male‐exposed and conspecific female‐exposed in K) vehicle‐treated VTA^GluCl*αβ*^ mice but not in L) IVM‐treated VTA^GluCl*αβ*^ mice (paired *t* test, *t*_5_(vehicle) = −4.205, *p* = 0.008, *t*_5_(IVM) = −2.239, *p* = 0.075). M,N) Correlations between [Low theta/High theta] ratios and total NREM sleep during recovery in M) vehicle‐ and N) IVM‐treated VTA^GluCl*αβ*^ mice (Spearman correlation). Data represent mean ± SEM.

What is the underlying mechanism driving the differential modulation of post‐SD sleep rebound by behavioral valence? Previous studies have shown that waking experience modulates sleep need^[^ ^43^, ^57^ ^]^ and that social stress like the one used in our CD‐1 male‐exposed mice increases sleep amount.^[^ ^58^, ^59^ ^]^ We therefore hypothesized that the SD protocols with either CD1‐male or a conspecific female induced different profiles of sleep pressure in animals. Surprisingly however, in vehicle‐treated VTA^GluCl*αβ*^ mice, the dynamic of NREM sleep delta (1–4 Hz) power after SD was not different in CD‐1 male‐exposed versus conspecific female‐exposed mice (**Figure** **7**A). Additionally, the overall NREM sleep delta power during recovery period was not different between CD‐1 male‐exposed and conspecific female‐exposed mice in vehicle‐treated VTA^GluCl*αβ*^ mice (Figure 7B). In IVM‐treated VTA^GluCl*αβ*^ mice, SD with CD‐1 male leads to significantly higher delta power during NREM sleep rebound relative to conspecific female‐exposed mice (Figure 7C,D) confirming their unaltered homeostatic regulation of sleep (Figure 2). No significant correlation was found between power density of NREM sleep delta waves and total amount of NREM sleep during recovery in both vehicle‐ (Figure S10A, Supporting Information) and IVM‐treated VTA^GluCl*αβ*^ mice (Figure S10B, Supporting Information). These results demonstrate that, in control animals, the modulation of sleep amount after SD by the valence of waking experience is mediated independently from SWA modulation. Following the inhibition of mDA neurons, sleep modulation by motivational valence is lost without affecting the homeostatic regulation of sleep.

The power density of other EEG frequencies during wakefulness have been shown to track sleep pressure.^[^ ^60^, ^61^, ^62^ ^]^ Additionally, because animals experience valence during wake states, we focused on spectral analyses of wake EEG during the two SD protocols. Interestingly, the two SD protocols induced distinct profiles of the EEG spectral power with specific valence‐related modulation at theta (4–12 Hz) range (Figure 7E). These results corroborate and extent a recent study linking theta‐rich wakefulness to motivated behaviors^[^ ^62^ ^]^ by revealing a differential modulation of low (4–8 Hz) and high theta (8–12 Hz) power densities by behavioral valence (Figure 7E). More specifically, we found that, relative to conspecific female‐exposed vehicle‐treated VTA^GluCl*αβ*^ mice, SD with CD‐1 male exposure induced a significant increase and decrease of the power density of, respectively, low theta (Figure 7E‐i) and high theta (Figure 7E‐ii). Consistent with recent evidence implicating DA in modulating high theta in the cortex,^[^ ^63^ ^]^ in IVM‐treated VTA^GluCl*αβ*^ mice, SD with CD‐1 male exposure failed to induce a significant decrease in high theta (Figure 7F‐ii). The power density of low theta still showed a significant increase in CD‐1 male‐exposed relative to conspecific female‐exposed VTA^GluCl*αβ*^ mice after IVM treatment (Figure 7F‐i). Consequently, we found significant negative correlation between high theta power density and total NREM sleep during recovery (Figure S10E, Supporting Information). Low theta power density did not correlate with the total amount of NREM sleep during recovery period (Figure S10C, Supporting Information). Both correlations are not significant following IVM treatment (Figure S10D,F, Supporting Information).

The discrimination between CD1‐male and conspecific female‐related SD protocols by high theta in vehicle‐treated VTA^GluCl*αβ*^ mice (Figure 7E‐ii) and its loss after IVM‐treatment (Figure 7F‐ii) is reflected more specifically in a narrower band of high theta (10.5–12 Hz) (Figure 7G–J). The power density of this narrower band was significantly lower during SD with CD‐1 male relative to SD with conspecific female in vehicle‐treated VTA^GluCl*αβ*^ mice (Figure 7G). No significant difference was found between the two SD protocols after IVM treatment (Figure 7H). Correspondingly, a significant negative correlation was found between the power density of the narrower band of high theta (10.5–12 Hz) and the total NREM sleep rebound following the two SD protocols (Figure 7I). This correlation is lost following IVM treatment in VTA^GluCl*αβ*^ mice (Figure 7J). Collectively, these results demonstrate that behavioral valence during sleep deprivation specifically modulates the power density of cortical theta (4–12 Hz) rhythms and that this modulation is significantly predictive of the subsequent NREM sleep rebound. Additionally, our results implicate mDA neurotransmission in the mediation of behavioral valence‐related modulation of both cortical theta and post‐SD sleep rebound.

### 2.8 Identification of a Potential EEG Marker for Valence‐Related Modulation of NREM Sleep Amount

Given the failure of the most reliable EEG marker for sleep depth (SWA) to mirror changes of NREM sleep amount induced by modulation of motivational drive (Figure 5 and Figure S5, Supporting Information) and behavioral valence (Figure 7A–D), we sought to identify a potential EEG marker for valence‐related modulation of the amount of NREM sleep. Because behavioral valence during SD differentially modulated low‐ and high‐theta power densities (Figure 7E,F), we computed ratios of power densities of [low theta/high theta] and compared them across the two SD protocols in both vehicle‐ and IVM‐treated VTA^GluCl*αβ*^ mice (Figure 7K–N). In vehicle‐treated mice, a significantly high [low theta/high theta] ratio was found during SD with CD‐1 male mice relative to SD with conspecific female mice (Figure 7K). Following IVM treatment, no significant difference of this ratio was found between the two SD protocols (Figure 7L). In addition, [low theta/high theta] ratio correlated positively in vehicle‐treated mice with total NREM sleep rebound following the two SD protocols (Figure 7M). This correlation flattened and lost its significance following IVM treatment (Figure 7N). These results show that the ratio of the power density of [low theta (4–8 Hz)/high theta (8–12 Hz)] during sleep deprivation is a reliable EEG index for tracking the modulation of NREM sleep rebound by behavioral valence.

## 3 Discussion

The necessary amount of sleep to maintain optimal performances is tightly regulated by the homeostatic process of sleep regulation.^[^ ^15^ ^]^ Yet, there is a large difference in the daily needed quota of sleep across species^[^ ^64^, ^65^, ^66^ ^]^ and even within the same species across different conditions.^[^ ^3^, ^4^, ^67^ ^]^ Although genetic factors play a prominent role in shaping sleep duration,^[^ ^64^, ^66^, ^68^ ^]^ genetics alone fail to account for the entire variability as well as the acute flexibility of the daily amount of sleep.^[^ ^69^ ^]^ In humans, chronic adoption of extreme sleep durations (≤4 or ≥10 h per night) is associated with global cognitive decline.^[^ ^70^, ^71^ ^]^ Similarly, chronic sleep deprivation in animals precipitates detrimental physiological responses that could lead to death.^[^ ^72^ ^]^ However, under motivationally charged conditions, several species can acutely adopt a regime of little or no sleep for remarkably long duration with surprisingly no degradation of performances and no subsequent sleep rebound.^[^ ^1^, ^2^, ^3^, ^4^, ^5^, ^6^, ^8^, ^9^, ^10^, ^11^, ^12^ ^]^ Conversely, stressful conditions are associated with robust alterations of sleep/wake cycle^[^ ^57^, ^72^ ^]^ and sleep rebounds following stress exposure are sometimes exaggerated.^[^ ^57^ ^]^ In addition of challenging the dominant paradigm of homeostatic sleep regulation,^[^ ^13^, ^14^ ^]^ the neural mechanism behind these remarkable adaptations and the motivational and stress‐related sleep modulation in general are unknown.

Building on the well‐established role of DA in encoding motivational and value‐related neural processing,^[^ ^16^, ^17^, ^18^, ^19^, ^20^, ^21^ ^]^ here, we examined the potential role of mDA in mediating motivation and valence‐related modulation of sleep/wake behavior as well as the mechanisms by which this modulation is achieved. Our study showed that, under baseline conditions, chronic silencing of DA neurons slightly increased NREM sleep (by ≈70 min) during the dark phase of the LD cycle. The manipulation of the contextual salience of the animals however revealed a strong deficit in eliciting adaptive arousal responses in face of salient stimuli leading to a more severe hypersomnia following DA inhibition. Surprisingly, the contextual modulation of NREM sleep by DA neurons inhibition was uncoupled from, and achieved without affecting, the homeostatic and circadian processes of sleep/wake regulation. We also found that the impacts of silencing DA neurons on the acute masking effects of light and dark could not account for the increased NREM sleep induced by DA neuron inhibition. In line with the role of DA in promoting and invigorating instrumental wakefulness,^[^ ^16^, ^17^, ^18^, ^19^, ^20^, ^21^ ^]^ we found that the sleep alterations induced by interfering with DA neurotransmission are rather due to a deficit in arousal induction which translates into a lower threshold of sleep initiation. Finally, we discovered a new role of DA in mediating valence‐related modulation of sleep. Collectively, our data suggest that mDA neurotransmission mediates a strong modulation of sleep/wake behavior by motivational and valence‐related factors which was uncoupled from the classical homeostatic and circadian processes of sleep regulation.

### 3.1 A Note on the Methodology used to Silence mDA Neurons

To selectively target and silence mDA neurons, we used the chemogenetic GluCl/IVM system. This system consists of a mutagenically modified chloride channel that lost its sensitivity to endogenous glutamate while retaining sensitivity to IVM.^[^ ^39^ ^]^ The GluCl*αβ* receptor is formed by a combinatory and simultaneous expression of two *α* and *β* subunits in the same neuron for its efficient and selective silencing. This system has also the advantage of inducing chronic inhibition lasting several days which make it, unlike the other chemo‐ and optogenetic systems,^[^ ^45^ ^]^ optimal for investigating sleep/wake and circadian rhythms. The efficiency of the GluCl/IVM system in inhibiting mDA neurons was confirmed in our study using double c‐fos/TH immunostaining. The c‐fos gene is a reliable marker for neural activity that has been used extensively to map neural networks activated by a broad range of stimuli^[^ ^128^ ^]^ including the DAergic reward pathways.^[^ ^129^ ^]^ The main limitation of the use of c‐fos as a proxy to neural activity is its low temporal resolution; c‐fos requires 45 to 90 min for its full expression making it therefore suboptimal relative to electrophysiology. This limitation is however not problematic at all with the use of the GluCl/IVM system that silences neurons for 3–4 days.^[^ ^39^ ^]^ The dramatic decrease of c‐fos expression in mDA neurons throughout the 2nd day following IVM treatment of VTA^GluCl*αβ*^ mice (Figure S3, Supporting Information) as well as all the behavioral phenotypes precipitated is reliable marker for the efficiency of the GluCl/IVM system to silence mDA neurons.

### 3.2 mDA Mediates Motivational Context‐Related Modulation of Sleep/Wake Cycle

The potential role of DA in modulating sleep/wake states dates back at least to the early studies identifying DA as an independent neurotransmitter in the brain.^[^ ^22^, ^23^ ^]^ During the ensuing decades, several lesioning, pharmacological, and genetic‐based studies in both humans and animal models, clearly identified DA as a potent wake promoting substance.^[^ ^24^, ^25^, ^26^ ^]^ However, the weak specificity of these early studies made it impossible to assess the relative role of DA versus other nonspecifically recruited neuronal pathways in the behavioral phenotypes induced.^[^ ^73^, ^74^, ^75^ ^]^ Additionally, the neuronal source of the effective DA remained unclear. The potential role of mDA neurons in sleep/wake regulation was masked for a while by early electrophysiological findings showing the lack of modulation of firing rates of DA neurons across sleep wake states^[^ ^76^, ^77^, ^78^, ^79^ ^]^ as well as by the revelation of a potent modulation of sleep/wake cycle by ventral periaqueductal gray (vPAG) DA neurons.^[^ ^29^, ^80^ ^]^ By using recently developed cell‐type‐specific optogenetic and chemogenetic approaches, recent work however has revealed a causal role of mDA neurons in inducing and maintaining electrocortical and behavioral arousal.^[^ ^27^, ^28^, ^29^, ^30^, ^31^, ^32^, ^33^ ^]^ The necessity of VTA DA neurons for behavioral arousal was also shown by chemogenetic^[^ ^27^, ^30^ ^]^ and optogenetic^[^ ^31^ ^]^ inhibitions that promoted sleep. Our study corroborates these findings by showing that chronic chemogenetic inhibition of mDA neurons induced a slight but sustained increase in NREM sleep over several days. This hypersomnia phenotype was restricted to the dark (active) phase of the animals, a finding that is consistent with the circadian increase of both VTA multiunit activity^[^ ^81^ ^]^ and DA concentration in neuronal targets of mDA innervation during the active phase.^[^ ^82^, ^83^, ^84^ ^]^

As a neuromodulator, DA impact on behavior^[^ ^48^ ^]^ as well as the recruited DAergic neurocircuitry is tightly modulated by the contextual salience.^[^ ^18^, ^47^, ^63^, ^85^ ^]^ Consistently, we found that the impact of silencing mDA on sleep/wake cycle was significantly dependent on the motivational salience of home environment. The most severe phenotypes were observed when mice were exposed to salient feeding and sexual stimuli (i.e., chocolate and female bedding). Facing these cues, control mice displayed strong adaptive arousal responses that are known to be essential for optimal behavioral performances. In contrast, following DA neurons inhibition, mice failed to generate and sustain such homeostatic responses. Our findings are consistent with previous work linking DA to motivational processes^[^ ^16^, ^17^, ^18^, ^19^, ^20^, ^21^ ^]^ and corroborate recent studies demonstrating the necessity of DA neurotransmission in maintaining wakefulness in arousal promoting environments.^[^ ^27^, ^29^, ^34^, ^35^ ^]^ Our study however extends these findings by showing that arousal deficits precipitated by the inhibition of mDA neurons are not restricted only to the time of exposure to salient environments but is maintained for several subsequent hours even after the end of the interaction with the salient stimuli. Our investigation of the 24 h sleep/wake behavior under several saliently different contexts allowed us also to appreciate the potential of contextual salience to modulate the outcome of silencing DA neurons on sleep/wake behavior. This finding could also explain the failure of few recent studies to detect significant alterations in sleep/wake parameters following the manipulation of mDA signaling^[^ ^34^, ^86^, ^87^ ^]^ although issues with the methodological approaches used to downregulate DA neurotransmission cannot be ruled out.^[^ ^45^, ^88^, ^89^, ^90^, ^91^ ^]^ As shown before,^[^ ^27^ ^]^ chronic silencing of mDA neurons did not affect the motivation as well as the skills of sleep‐related nest building behavior. Combining our results with previous work suggests therefore a primordial role of mDA neurons in mediating adaptive modulation of sleep/wake behavior by contextual salience.

### 3.3 Inhibition of DA Neurons Promotes Sleep without Affecting Sleep Pressure and the Homeostatic Process of Sleep/Wake Regulation

By what mechanism sleep is promoted following silencing of mDA? Because sleep duration and intensity are homeostatically regulated.^[^ ^13^, ^14^ ^]^ We first examined the impact of DA neurons inhibition on the depth of sleep and its homeostatic regulation. Surprisingly, silencing mDA neurons did not increase sleep pressure during wake states, nor did it affect the homeostatic regulation of sleep following SD. This neurophysiological profile is unique and departs from the impacts of downregulating other wake‐promoting neuromodulators on sleep/wake behavior as well as the mechanism by which they mediate their effects. Synaptic inhibition of Ach neurons in the basal forebrain or knockout of Ach receptors induces chronic decrease of NREM and REM sleep.^[^ ^92^ ^]^ Similarly, ablation of serotonin neurons increases wakefulness and impair the homeostatic response to SD.^[^ ^93^ ^]^ Genetic deletion of orexin peptides or receptors induce severe fragmentation of sleep/wake cycle without affecting quantitative aspects of sleep/wake states, or sleep need and its homeostatic regulation.^[^ ^94^ ^]^ Behavioral activation by salient stimuli is also unaffected in orexin knockout mice.^[^ ^94^ ^]^ The promotion of sleep by melanin‐concentrating hormone neurons activation in the LH is mediated by increased sleep pressure as evidenced by significant increase in SWA.^[^ ^95^ ^]^ A similar mechanism accounts for the severe hypersomnia in *Sleepy* mice harboring a splicing mutation in the Sik3 protein kinase gene.^[^ ^64^ ^]^ Like in Orexin knockout mice, Sleepy mice showed a normal arousal response to salient stimuli.^[^ ^64^ ^]^ Perhaps the closet sleep/wake profile to the one displayed by our animals following mDA neurons inhibition is found in mice lacking histamine or norepinephrine (NE). Downregulation of these two wake‐promoting neurotransmitters is also associated with a slight but significant increase of NREM sleep during the dark phase without a concomitant increase in SWA.^[^ ^96^, ^97^, ^98^ ^]^ Additionally, and like our data, mice deficient of histamine or NE lack the adaptive behavioral attention to salient environments.^[^ ^96^, ^97^, ^98^ ^]^ Notable, silencing other DA neurons in vPAG increases NREM sleep and impairs arousal responses to salient stimuli through prominent increase of sleep and SWA.^[^ ^29^ ^]^ Conversely, stimulation, rather that inhibition, of DA fibers originating from substantia nigra in the dorsal striatum increases both NREM sleep and SWA.^[^ ^32^ ^]^ Taken together, these studies indicate that although the wake promoting neuromodulators are recruited during wake states, they are functionally and mechanistically heterogenous and not necessarily redundant in their mediated physiological and behavioral profiles.

If downregulation of mDA neurotransmission promotes sleep without affecting the homeostatic process of sleep regulation, what about its upregulation? As mentioned in the introduction, interventions that upregulate synaptic DA concentration strongly promote wake.^[^ ^24^ ^]^ Genetic deletion of DAT does not change baseline DA levels but slow down its clearance from the extracellular space.^[^ ^99^ ^]^ The induced hyperdopaminergic phenotype is associated with a prominent decrease in sleep duration and a threefold increase in wake bout duration.^[^ ^25^ ^]^ Few recent optogenetic and chemogenetic studies have also examined sleep/wake after DA upregulation induced by selective inhibition of midbrain gamma‐aminobutyric acid (GABA) neurons.^[^ ^46^, ^86^, ^100^, ^101^, ^102^, ^103^ ^]^ This GABAergic neural population accounts for 30% of total VTA and SN neurons and exerts a powerful inhibition on neighboring DA neurons.^[^ ^104^, ^105^ ^]^ Their inhibition dramatically decreases sleep amount and increases wakefulness.^[^ ^46^, ^86^, ^100^, ^101^, ^102^, ^103^ ^]^ Two mechanisms could account for this phenotype. DA could either prevent the build‐up of sleep pressure or just antagonize it. Evidence for the later mechanism has been provided recently by Honda et al.^[^ ^101^ ^]^ By examining the homeostatic response to SD following ablation of midbrain GABA neurons, the authors found that despite lacking sleep rebound, mice still showed the typical increase and dissipation of SWA that was indistinguishable from control mice.^[^ ^101^ ^]^ These results demonstrate that increased DAergic tone does not interfere with the homeostatic processes of sleep regulation but masks its manifestation by independently antagonizing its action. The recent identification of wake^[^ ^35^ ^]^ and sleep‐promoting medium‐spiny neurons in the striatum^[^ ^34^ ^]^ that are sensitive and antagonistically modulated by DA and adenosine^[^ ^106^ ^]^ adds to the indirect evidence from human studies^[^ ^107^ ^]^ that points to ventral striatum as the critical downstream neuronal structure mediating the modulation of sleep by mDA neurotransmission. Combined, our data and literature evidence demonstrate that up‐ and downregulation of mDA signaling strongly mediates motivational modulation of sleep/wake behavior either by overriding or without affecting the homeostatic process of sleep regulation.

### 3.4 DA Neurons Inhibition Does Not Affect the Central Circadian Clock

The second pillar of the two‐process model of sleep regulation is governed by the circadian pacemaker. Although initially, the circadian process was thought of as essential in orchestrating the qualitative timing aspects of sleep/wake states,^[^ ^13^ ^]^ subsequent evidences have causally implicated clock genes in sleep homeostasis.^[^ ^15^ ^]^ In fact, two recent studies have provided strong evidence implicating directly the central circadian clock in modulating quantitative aspects of sleep.^[^ ^51^, ^52^ ^]^ Additionally, selective activation of DA neurotransmission in the SCN has been shown to effectively modulate circadian rhythms.^[^ ^108^, ^109^, ^110^ ^]^ We therefore examined the impact of silencing midbrain DA neurons on the two fundamental properties of the circadian clock, i.e., the endogenous period and photoentrainment. Our results show that both properties are fundamentally intact suggesting therefore that sleep phenotypes induced by DA neurons inhibition could not be attributed to a dysfunctional central clock. This conclusion is further supported by several studies showing normal aspects of several physiological and molecular markers of the central clock following intervention that downregulate DA neurotransmission in flies,^[^ ^111^, ^112^ ^]^ mice,^[^ ^83^ ^]^ nonhuman primates,^[^ ^113^ ^]^ and even patients with Parkinson's disease.^[^ ^114^, ^115^ ^]^

### 3.5 The Acute Masking Effects of Light and Dark on Sleep/Wake Behavior Do Not Account for Sleep Alterations Induced by Inhibition of mDA Neurons

A recent study found that ablation of VTA DA neurons abolishes the acute wake promoting effect of darkness.^[^ ^87^ ^]^ We therefore investigated the potential of this pathophysiological mechanism in contributing to sleep/wake alterations induced by inhibition of DA neurons. Using two light exposure protocols, our data show that, although occasionally mice displayed altered masking responses, over 24 h day the sensitivity and modulation of sleep/wake states by light and dark is not altered after inhibition of DA neurons. These findings are again corroborated by studies in fruit flies,^[^ ^111^, ^112^ ^]^ mice^[^ ^41^ ^]^ and nonhuman primates^[^ ^113^ ^]^ showing normal masking responses to light and dark following selective lesion of, or blockade of synaptic neurotransmission in, DA neurons. We therefore conclude that the hypersomnia induced by silencing DA neurons is not mediated by dysfunctional masking responses to light and darkness.

### 3.6 Inhibition of mDA Neurons Impairs Instrumental Wakefulness

Wake state encompasses a wide spectrum of arousal levels that are supervened upon different neuronal dynamics across different behavioral contexts.^[^ ^116^ ^]^ In line with recent studies implicating mDA in encoding the value of work and in mediating instrumental wakefulness,^[^ ^16^, ^17^, ^18^, ^19^, ^20^, ^21^ ^]^ we found a severe deficit in maintaining adaptive arousal in face of salient stimuli after inhibiting mDA neurons. This deficit was translated into lower thresholds of inducing sleep as evidenced by shorter sleep latencies and was exacerbated by increasing sleep pressure through SD. Given that this arousal deficit occurs within a physiological background of normal homeostatic regulation of sleep pressure, our data corroborate our previous study showing that homeostatic sleep need and arousal are regulated independently.^[^ ^43^ ^]^ Our study also demonstrates that, during wakefulness, under high sleep pressure, arousal factors can be mobilized and effectively antagonize and overcome homeostatic factors driving sleep. Although arousal level is considered to reflect the sum of all wake‐promoting neuromodulators in the brain,^[^ ^55^ ^]^ not all of them are required for instrumental wakefulness. Deletion of orexin, e.g., does not impair arousal responses to salient environments.^[^ ^94^ ^]^ Our results strongly implicate mDA in mediating these adaptive responses and indicate that, under motivational contexts, appropriate arousal is conditioned upon the activation of mDA neurotransmission. Under similar circumstances, other neurotransmitters such as histamine^[^ ^96^ ^]^ and NE^[^ ^98^ ^]^ might also be involved. Future studies should investigate the relative contribution of different wake‐promoting neuromodulators to motivationally driven wakefulness and whether their action is, like mDA, uncoupled from the homeostatic and circadian processes of sleep/wake regulation.

### 3.7 Midbrain DA Mediates Valence‐Related Modulation of Sleep/Wake Behavior

Contextual valence is a powerful factor that shapes adaptive behavior responses.^[^ ^116^ ^]^ The functional relationship between emotional valence and sleep is bidirectional.^[^ ^57^, ^72^, ^107^ ^]^ While the mechanisms by which sleep affects emotional processing have started to be elucidated,^[^ ^107^ ^]^ the neuronal mechanisms by which emotional valence shapes qualitative and quantitative aspects of sleep are still unknown. As discussed in the introduction, several recent studies have revealed intriguing examples of extreme, but adaptive loss of sleep with minimal or no subsequent sleep rebound in several species under emotionally charged conditions (i.e., sexual activity, reproductive migration).^[^ ^1^, ^2^, ^3^, ^4^, ^5^, ^6^, ^8^, ^9^, ^10^, ^11^, ^12^ ^]^ Conversely, sleep loss under stressful conditions can lead to exacerbated sleep rebound.^[^ ^57^, ^58^ ^]^ Our study identifies mDA neurotransmission as an important neural substrate mediating valence‐related modulation of sleep. By using two protocols of sleep deprivation during the same circadian time, but with opposite motivational valences, we show that in control mice social stress‐induced SD leads to stronger sleep rebound compared to conspecific female‐induced SD. A differential stress response in terms of HPA (hypothalamic–pituitary–adrenal) axis activation between the two SD protocols is unlikely to account for the different sleep rebound phenotypes. We^[^ ^43^ ^]^ and others^[^ ^57^, ^59^ ^]^ have shown that corticosterone concentration, as a marker of HPA axis activation, during different SD protocols, are dissociated from, and do not correlate with subsequent sleep rebound responses. Additionally, first sexual interaction (like we used in female‐induced SD) is known to induce dramatic increases in blood levels of corticosterone, epinephrine, and norepinephrine in male mice as well.^[^ ^117^, ^118^ ^]^ The degree of behavioral arousal in the SD conditions is unlikely as well to account for the differences in sleep rebound. In both conditions, mice displayed high levels of behavioral activation. Interestingly, sleep rebound was stronger in CD1‐male‐exposed‐mice despite losing relatively less sleep during SD. This finding is the opposite of what we would expect if the degree of behavioral activation is the main driver of the observed sleep rebound. Additionally, the power density of theta (6–9.5 Hz) throughout SD was undisguisable between the two SD protocols. This theta‐dominated wakefulness has been recently associated with the degree of behavioral arousal linked with motivated behaviors.^[^ ^62^ ^]^ Taken together therefore, the levels of both HPA axis and behavioral activations cannot account for the different intensities in sleep rebound following extended wakefulness induced by opposite motivational valences.

The most parsimonious explanation of the different sleep rebound phenotypes induced by the two SD protocols is that the valence of SD experience itself differently modulates sleep/wake processes. This explanation is corroborated by the impairment of the differential modulation of sleep rebound by the two SD protocols following inhibition of mDA neurons. These findings are also in line with the well‐established role of mDA neurons in encoding valence.^[^ ^17^, ^116^ ^]^ In addition to mDA neurotransmission, several other brain areas including, ventral striatum, basolateral‐amygdala, ventral pallidum, prefrontal cortex, and lateral hypothalamus are known to encode behavioral valence (reviewed in ref. [116]). Interestingly, all of these neural centers are innervated and modulated by mDA neurons^[^ ^116^, ^119^ ^]^ and have been recently involved in gating the modulation of sleep/wake cycle by motivational behaviors.^[^ ^34^, ^35^, ^120^, ^121^ ^]^ This motivational and valence‐encoding neuronal network is therefore well suited to mediate the modulation of sleep by behavioral valence. How is this valence ‐related modulation of sleep encoded and tracked over time will be the focus of intense future investigations.

### 3.8 A Potential EEG Marker for Valence‐Dependent Modulation of Sleep

Surprisingly, no difference in the dynamic and magnitude of SWA during NREM sleep following SD was found between the two SD protocols. This finding corroborates our earlier results (Figure 2 and Figure S6, Supporting Information) and demonstrates that not only motivation but also motivational valence modulates sleep amount without affecting the homeostatic process of sleep regulation. Gold standard EEG markers of homeostatic sleep pressure are therefore unlikely to track sleep modulation by motivational valence. Interestingly, comparable dissociations between SWA and sleep amount have occasionally been reported whenever motivational valence of wakefulness was manipulated.^[^ ^4^, ^5^, ^43^, ^59^, ^122^, ^123^ ^]^ Because emotional valence is consciously experienced mainly during wake states, and given the high fidelity of EEG to reflect the wide spectrum of arousal levels, sensorimotor processing modes, and behaviors associated with different wake states,^[^ ^116^ ^]^ we analyzed the spectral composition of EEG during SD and compared it between CD‐1 male and conspecific female‐exposed mice. We were able to identify a reliable EEG index at the theta range (i.e., low theta/high theta) that significantly and positively correlated with valence‐modulated sleep amount. Interestingly, and consistent with the mediation of motivational valence‐modulation of sleep by mDA signaling, the identified EEG marker was sensitive to the inhibition of DA neurons. Notably, the identification of the most reliable EEG marker within theta range corroborates the recently identified theta‐dominated wakefulness that underly motivated behaviors.^[^ ^62^ ^]^ Furthermore, mDA is known to significantly modulate theta activity^[^ ^124^ ^]^ and a recent report showed that this modulation is contextual^[^ ^63^ ^]^ reinforcing the potential of theta activity to be modulated by, and reliably reflect, contextual valence. Because theta oscillations are known to be modulated by a wide range of cognitive and behavioral parameters,^[^ ^125^, ^126^ ^]^ we would like to emphasize that probably not any theta activity would reliably track valence‐related modulation of sleep. Rather, as we show here, theta activity that dominates valence‐charged wake states would be more reliable and faithful in tracking sleep/wake modulation by motivational valence. Future studies using high density and high‐resolution local field potentials recordings combined with specific modulation of contextual valence are expected to further characterize the origin and specificity of theta and other frequency oscillations in encoding and tracking sleep changes linked to behavioral valence.

### 3.9 Conclusions and Translational Relevance

In summary, at the fundamental level, our study provides the first demonstration that mDA neurotransmission mediates the modulation of sleep/wake behavior by motivational valence. This modulation is uncoupled from the classical circadian and homeostatic processes of sleep regulation which establishes the foundation for a new factor of sleep regulation (which we may call factor V). From a translational perspective, our findings are expected to provide useful insights for the development of efficient therapeutic strategies against intrinsic sleep/wake disorders and sleep/arousal alterations associated with several neurological and neuropsychiatric diseases with dysfunctional DA signaling.

## 4 Experimental Section

### Experimental Model and Subject Details

DA transporter (DAT)‐Cre knock in mice were obtained from the Jackson Laboratory (# 006660) and crossed with wild‐type C57BL/6J mice. For all experiments, male mice were exclusively used. After weaning, animals were group‐housed until they were 12–20 weeks old. Upon the completion of any surgical procedures, mice were allowed to recover from anesthesia on a heating‐pad, and then they were transferred to their residence room for full recovery (at least 1 week) prior to the start of behavioral experiments or habituation to EEG/EMG tethers. Animals were housed on a 12 h/12 h light–dark cycle (lights on at 9 AM, lights off at 9 PM) with ad libitum access to food and water. Animal husbandry and experimental procedures were performed in accordance with the Animal Care Committee of the University of Tsukuba (approved protocol ID #180094). Extra effort was made to minimize the number of animals used as well as any stress or discomfort.

### Surgeries for Viral Delivery and EEG/EMG Implantation

*General Surgical Procedures and Viral Injections*: For midbrain microinjections, mice were anesthetized with isoflurane gas/carbogen mixture (3% for induction and 1.5–2% for maintenance during experimental surgery) and placed to a mouse stereotaxic apparatus (David Kopf Instruments, CA, USA). Mouse's head was shaved, and the skin was sterilized with betadine (Povidone‐iodine), then a midline incision was made with sterile scissors. The surface of the skull was scratched and cleaned with autoclaved cotton swabs. Bregma and Lambda were leveled to be on the same *z*‐axis. Small craniotomy holes were made for viral injection over the VTA (antero‐posterior (AP) axis: −3.4 mm, medio‐lateral (ML) axis: ±0.38 mm). AAV vectors (see below for details) were injected bilaterally into the VTA (AP: −3.4 mm, ML: ±0.38 mm, dorso‐ventral (DV): −3.8 mm). Viral injections were performed using a glass micropipette connected to an air pressure controller system (Picospritzer III, Parker Hannifin Co.). 0.4–0.5 µL of AAV was injected per site at a rate of 10 nL min^−1^. Following the injection, the micropipette was held in the same position for an additional 10 min to allow sufficient diffusion. Another 5 min was used to withdraw the micropipette as to prevent potential backflow over micropipette's track.

*EEG And EMG Implantation*: Mice were chronically implanted with EEG and EMG electrodes for polysomnography. For this, three additional craniotomy holes were made in frontal (for EEG channel: AP: +1.5 mm, ML: +1 mm) and parietal (for reference and ground; AP: −3.5 mm, ML: 2.5 mm) regions. The third craniotomy hole was made in lateral parietal regions and was used for implant support. Two insulated, Teflon‐coated silver wires were inserted bilaterally into the trapezius muscles and served as EMG electrodes. The EEG/EMG electrodes were fixed to the skull using dental cement and then the incision was closed.

*Plasmids and Viral Constructs*: AAV serotype 10 encoding *α* subunit of the IVM‐gated chloride channel *α*‐optGluCl (39) under EYFP promotor and CRE‐dependent FLEX switch (AAV10‐FLEX‐*α*‐optGluCl‐EYFP, 9.24 × 10^13^ genome copies mL^−1^) and an AAV serotype 10 encoding *β* subunit *β*‐optGluCl under ECFP promoter (AAV10‐FLEX‐*β*‐optGluCl‐ECFP, 1.16 × 10^14^ genome copies mL^−1^) were bilaterally injected. Experimental group received a mixture of both virus while control animals received only the AAV virus encoding for the *β* subunit. The viruses were generated by tripartite transfection (AAV‐rep2/caprh 10 expression plasmid, adenovirus helper plasmid, and pAAV‐FLEX‐*α*‐optGluCl‐EYFP or pAAV‐*β*‐optGluCl‐ECFP plasmid) into 293A cells. Plasmids were thankfully donated by Dr. Patrick M Fuller. After 3 days, 293A cells were resuspended in artificial cerebrospinal fluid (aCSF), freeze‐thawed four times, and treated with benzonase nuclease (Millipore) to degrade all forms of DNA and RNA. Subsequently, centrifugation was used to remove cell debris and the virus titer in the supernatant was determined using by quantitative polymerase chain reaction. Aliquots of virus were stored at −80 °C before stereotaxic injection.

### Experimental Design

All behavioral and sleep‐related experiments were performed at least 4 weeks after surgery to allow for maximal transgene expression. Mice underwent the following assays in their sleep recording chambers, unless otherwise stated.

*Validation of the Efficiency of GluCl/IVM Chemogenetic System*: To confirm the efficiency of GluCl/IVM in silencing midbrain DA neurons, neural activity of DA neurons over 24 h 1 day after IVM treatment of VTA^GluCl*αβ*^ mice was assessed. Starting from CT0, brain tissues were collected from three mice every 3 h. The same protocol was followed for vehicle‐treated VTA^GluCl*αβ*^ mice for control reference. The expression of c‐fos was used as a marker of neural activity while the expression of TH was used to identify midbrain DA neurons (see Histology section). During this experiment, mice were housed in their home cages under constant darkness conditions.

*Sleep/Wake Vigilance States Recording*: Individually housed mice were introduced to sleep chambers and connected to EEG/EMG recording cables. Animals were habituated to sleep chambers for 2–3 consecutive days under 12 h/12 h LD cycle. During this habituation phase, animals were handled every day for ≈5 min at the beginning of the light phase in order to prepare them for intraperitoneal (i.p.) injections of vehicle (propylene glycol) or IVM. Polysomnography recordings subsequently stated at the beginning of light phase and lasted for as long as required depending on the assays (see below).

*Salient Stimuli Exposure Assays*: After habituation, 24 h baseline EEG/EMG recording was performed. Animal were then exposed for 1 h at the beginning of the light phase (ZT0‐1) and for another 1 at the beginning of the dark phase (ZT12‐13) to one of the following salient objects; white chocolate, snake skin shed, a familiar squared object, or fresh female beddings. The white chocolate consisted of small, equally sized pieces (1 cm, 1 cm) and was purchased from Meiji holdings Co, Japan. The rectangular object (3 cm^3^) was made familiar to animals by introducing it into their home cage white Kleenex tissue as an enrichment during habituation phase. The squared object was removed from the cage 1 day before its introduction again during the assays. Female beddings consisted of 1 g of a fresh mixture of bedding and tissue from a cage inhabited by five young conspecific females (12–20 weeks old). The bedding was introduced in 1.5 mL Eppendorf tube that were perforated with small holes in order to allow for easy access to odors. All stimuli were removed from animal's home cage 1 h after introduction both during the light and dark phase assays. EEG/EMG recordings continued until all salient stimuli exposure assays ended.

*Nest‐Building Assay*: In the nest‐building experiment presented in Figure 1L–O, mice were injected with either vehicle or IVM (5 mg kg^−1^) at the beginning of the light phase (ZT0). 24 h after the injections, the old nest was removed from the home cage and new nesting material (3 g) was introduced. Nest‐building behavior was evaluated 4 h later (at ZT4). EEG/EMG data were recorded simultaneously during this assay.

The evaluation of nest‐building was based on Deacon in 2006 using a five‐point scale:^[^ ^127^ ^]^

1) Nesting material was not noticeably touched (more than 90% intact).
2) Nesting material was partially torn (50–90% remaining intact).
3) Nesting material shredded and torn up but without an identifiable nest site: less than 50% of the nesting material remained intact, but less than 90% was within a quarter of the cage floor area, i.e., the tissue was not gathered into a nest but was spread around the cage.
4) A clearly identifiable but flat nest; more than 90% of the nesting material was shredded and gathered into a nest within a quarter of the cage floor area, but the nest was flat, with walls higher than mouse body height (of a mouse curled up on its side) for less than 50% of its circumference crater. Walls of the nest were higher than mouse body height for more than 50% of its circumference.

*4 h Sleep Deprivation Assay*: Mice were sleep deprived for 4 h starting from light's on using enriched, novel environment in order to stimulate spontaneous exploratory wake states. This method was previously validated and was shown to not increase plasma corticosterone, hence not producing significant negative stress in mice.^[^ ^40^ ^]^ Clean bedding, food, water, toys, and novel nesting tissue were used to stimulate motivational wake. Mice were continuously monitored by an experimenter (FK or AEF) via their online EEG/EMG signals. Whenever mice appeared to be entering NREM sleep (i.e., noticeable increase in slow wave amplitude), new material was introduced to the cage of the animal. Touching animals directly was avoided to limit confounding stress.

*Caffeine Injection Assay*: For the caffeine injection experiment depicted in Figure 2H–L, 24 h following vehicle or IVM (5 mg kg^−1^) treatment, VTA^GluCl*αβ*^ mice were injected i.p. with saline or 15 mg kg^−1^ of caffeine. Caffeine was dissolved in sterile 0.9% saline. EEG/EMG signals were recorded simultaneously for 2 days (1 day before and 1 day after saline or caffeine injection).

*Circadian Rhythm Evaluation Assay*: Circadian rhythms were assessed by monitoring rest/activity cycles using previously described methods.^[^ ^41^ ^]^ Animals were individually housed in new cages and introduced into locomotor activity recording chambers in a new experimental room. Cages were equipped with passive IR motion captors and data were recorded using a computerized data acquisition system (Med Associates Inc., USA). Mice were acclimatized to the new environment for at least 3 days before the data acquisition was started. Vehicle‐treated VTA^GluCl*αβ*^ were first exposed to a 12 h light:12 h dark cycle (LD) for 1 week to measure daily rest/activity rhythms and photoentrainment. At ZT14 (2 h after activity onset) of the last day of LD assay, mice were exposed to a light pulse of 15 min (white fluorescent light, 300 lux), then released into constant darkness (DD) for the following 12 days in order to assess endogenous properties of the rest/activity rhythm. The same LD cycle, light pulse exposure, and DD assays were then repeated in the same animal under IVM (5 mg kg^−1^) treatment. During these assays, vehicle and IVM i.p. injections were done every 3 days at randomly selected time in order to avoid potential interference with behavioral rhythms. Red light was used for visualization during the injections that occurred while the animals were in darkness.

For circadian data analyses, Wheel Analysis #SOF‐861 (Med Associates Inc., USA) was used. Photoentrainment to LD cycle was estimated by computing phase angle of entrainment defined as the mean of the difference between light‐off and activity onset. The Chi‐squared periodogram method^[^ ^42^ ^]^ was used to calculate the period of the behavioral rhythm which was defined as the time taken for one complete cycle (i.e., the time between two consecutive peaks or troughs of a recurring rhythm). The subjective day and subjective night were defined as the segment of a circadian cycle during the free running condition that corresponded to, respectively, the light phase and dark phase of the LD cycle. Activity onset which was a robust indicator of entrainment corresponded to the average clock (or circadian) time of activity onset. Regression lines through activity onsets before and after light pulse were used to calculate the phase shift. Finally, the amplitude of the rest/activity rhythms, defined as peak‐to‐nadir difference was extracted from the peak of the Chi‐squared periodogram.

To assess directly if the central SCN clock was affected following the inhibition of midbrain DA neurons, the circadian pattern of SCN activity was examined using c‐fos as a marker. One day following IVM or vehicle treatment of VTA^GluCl*αβ*^ mice, three mice were sacrificed every 3 h over 24 h for both IVM‐ and vehicle‐treated VTA^GluCl*αβ*^ mice. Brains were collected and processed for c‐fos immunostaining.

*SCN Sensitivity to Light Assay*: To assess whether SCN sensitivity to light was affected by silencing midbrain DA neurons, well‐entrained IVM‐treated VTA^GluCl*αβ*^ mice (*n* = 6) were exposed to 1 h of light (300 lux) at ZT14‐15. At the end of light exposure, animals were sacrificed and brains were collected for immunohistochemistry. Vehicle‐treated VTA^GluCl*αβ*^ mice (*n* = 6) that were entrained to 12 h/12 h LD cycles were sacrificed at ZT15 without light exposure and were used as baseline controls.

*Masking Assays*: While mice were connected to sleep chambers, and simultaneous of EEG/EMG recording, two protocols were used to assess masking responses to light and dark pulses. The first protocol was consisted of exposing VTA^GluCl*αβ*^ mice to 1 h light and 1 h dark pulses, respectively, ZT14‐15 and ZT2‐3 following both vehicle and IVM (5 mg kg^−1^) treatment. Percentages of vigilance states during light and dark pulses were compared with those of the same time interval on the preceding baseline day for each animal. The second protocol was consisted of exposing animals to a T2 light/dark cycle (ultradian, 1 h; 300 lux; 1 h dark) for 24 h. Total amount of sleep and wake as well as their relative distribution within, respectively, light and dark pulses was computed.

### Arousal Responses under Different Conditions of Sleep Pressure Test

To assess arousal responses of mice under different levels of sleep pressure (Figure 6), vehicle‐ and IVM (5 mg kg^−1^)‐treated mice were subjected to increasing durations of sleep deprivation (1 to 6 h) using the same methodology which was adopted for the 4 h SD assay. At least 4–5 days separated successive SD experiments. 30 min after the end of SD, fresh female bedding was introduced into animal's home cage for 1 h. Total amount of each vigilance states during this hour as well as sleep latency was calculated. Sleep latency was defined as the elapsed time between female bedding introduction and the initial appearance of NREM sleep throughout a 20 s epoch.^[^ ^43^ ^]^

### Valence‐Related Sleep Deprivation Experiments

To sleep deprive animals with opposite valence‐charged wake experiences, individually housed VTA^GluCl*αβ*^ mice following vehicle and IVM (5 mg kg^−1^) treatment to two different SD protocols were subjected. The negatively charged SD protocol was consisted of an acute social defeat stress with aggressive CD‐1 male mice. To screen for aggressive intruder mice, male CD‐1 mice (body weight >40 g) were individually housed and trained to display aggression against male C57BL/6J mice by placing a male C57BL/6J mouse in the cage of a CD‐1 mouse one or two times a week. During training, C57BL/6J mouse was quickly removed from the cage as soon as it was attacked and defeated by CD‐1 mouse. Unsuitable CD‐1 mice that showed little aggression or extreme violent behavior were excluded from the experiment. CD‐1 mice that successfully attacked C57BL/6J mice within 1 min of introduction were used in the experiment to ensure successful social defeat stress.

The social defeat trial lasted for 10 min and started by introducing a CD‐1 mouse into the home cage of VTA^GluCl*αβ*^ mouse. Direct contact was allowed during the first 5 min during which VTA^GluCl*αβ*^ mice were typically attacked three to four times. During the last 5 min, CD‐1 male was isolated in rectangular wire‐mesh box to limit further physical attacks without preventing olfactory, visual, and auditory contacts. At the end of 10 min test, CD‐1 male mouse was removed from the cage of VTA^GluCl*αβ*^ mouse. This procedure was repeated four times at the start of each hour during the 4 h SD experience. At the end of the SD, any feces left by CD‐1 male mouse were removed from the cage and VTA^GluCl*αβ*^ mice were left undisturbed for recovery.

The positively charged SD protocol was consisted of introducing a virgin C57BL/6J female mouse to VTA^GluCl*αβ*^ mice during the 4 h of SD. For this experiment, 3–5 months old C57BL/6J females were used that were group housed (4–5 mice per cage) since weaning. No attempt was made to verify the stage of the estrous cycle of mice. C57BL/6J female mouse was introduced into the cage for direct and free contact with VTA^GluCl*αβ*^ mice for 30 min, then removed from the cage for the remaining 30 min every hour. This cycle was repeated four times during the 4 h SD experience. At the end of the SD, any feces left by female mice were removed from the cage and VTA^GluCl*αβ*^ mice were left undisturbed for recovery. Female bedding was not used for these SD experiments even though it was perceived positively by VTA^GluCl*αβ*^ mice because it was not efficient enough to achieve >90% sleep loss over 4 h. The use of virgin female mice achieved this goal without potential confounding stress. To control for potential side effects of IVM treatment, wild‐type mice were treated with IVM (i.p. 5 mg kg^−1^) and subjected to the same positively and negatively charged SD protocols (Figure S9, Supporting Information).

### EEG/EMG Data Acquisition, Sleep/Wake Vigilance State Determination, and Data Processing and Analysis

After EEG/EMG implantation surgery and recovery period, animals were connected through a tether and a home‐made commutator into the recording set‐up. EEG/EMG signals were amplified and filtered (0.65 Hz high‐pass filter for EEG, 5–30 Hz band‐pass filter for EMG), digitized with a sampling rate of 128 or 256 Hz and recorded using sleep analysis software (SleepSign for animal, Kissei Comtec CO., Nagano, Japan).

Sleep/wake states were first automatically scored offline in 10 s epochs by SleepSign software. Polysomnographic recordings were then reloaded, visually examined, and manually corrected when necessary. Classification of vigilance states was performed according to established criteria into three states; 1) wakefulness characterized with desynchronized, low‐amplitude, and high‐frequency EEG signals and increased EMG activity, 2) NREM sleep defined as a sleep state with synchronized, high‐amplitude, and low‐frequency (<4 Hz) EEG signals with decreased EMG activity, and 3) REM sleep with its characteristic high theta oscillations in the EEG signals and strongly reduced EMG activity.

To visualize spectral properties of EEG signals across distinct vigilance states, EEG was decomposed into time‐frequency domain using fast Fourier transform (FFT) at the frequency range 0.20 Hz for all experiments except the valence related SD (Figure 7) for which a broader frequency range (0–80 Hz) was analyzed with a resolution of 0.5 or 1 Hz. Relative power bands were summed as follows; delta (1–4 Hz), low theta (4–8 Hz), high theta (8–12 Hz), beta (12–30 Hz), and Gamma (30–80 Hz). The last two bands were mainly relevant for valence‐related SD experiments (Figure 7). Every 10 s, epoch of EEG power spectra was analyzed by FFT. The power spectra were normalized by computing the percentage of each 0.5 or 1 Hz bin from the mean of the baseline for each individual animal. This computation was done in a state‐dependent manner; the power of each bin was first averaged for each specific stage (wake, NREM sleep, and REM sleep) individually, then normalized as a group by calculating the percentage of each bin from the mean of the baseline of the individual animal.

Because the percentage of sleep lost during valence‐related SD experiments (Figure 7) was slightly different in CD‐1‐exposed relative to conspecific‐female‐exposed VTA^GluCl*αβ*^ mice, SD responses were normalized as the ratio of recovery NREM sleep for each animal over sleep lost during SD. Sleep lost was defined as the difference between total NREM sleep during corresponding baseline day and total NREM sleep still displayed during SD experiment:

SD response = [Recovery NREM sleep/Lost NREM sleep] = [Recovery NREM sleep/(Baseline NREM sleep−SD NREM sleep)].

### Histology

*Perfusion*: Under deep anesthesia, mice were transcardially perfused with saline then with 4% paraformaldehyde (PFA) in phosphate‐buffered saline (PBS). Mouse brains were carefully removed from the skull and post‐fixed in 4% PFA at 4 °C. Brains were then switched to 20% sucrose over 2 days at 4 °C, then sectioned into 40 µm coronal slices with a freezing microtome (Thermo Scientific, Cryostat, NX70, USA). Slices were stored in PBS at 4 °C until immunohistochemical processing.

*Immunohistochemistry*: Brain sections were incubated in 0.3% hydrogen peroxide and then overnight in a PBS solution containing 0.1% Triton X‐100 and primary antibodies (see below for details) at 4 °C. Afterward, sections were washed three times (10 min each) in PBS. For staining that was revealed by DAB/H_2_O_2_, sections were then incubated for 2 h in biotinylated antibody (1:1000, Jackson ImmunoResearch Laboratories). Sections were again washed by PBS solution three times (10 min each) then treated with avidin‐biotin complex (1:1000, Vectastain ABC Elite kit, Vector laboratories) for 1 h. After another round of three successive washing by PBS (10 min each), staining was visualized by monitored reaction with 3,3’‐diaminobenzidine and 0.01% hydrogen peroxide. For the double c‐fos/TH immunostaining, the first revelation was performed using 3,3’‐diaminobenzidine and 0.01% hydrogen peroxide to which 0.01% nickel ammonium sulfate and 0.005% cobalt chloride were added. This led to a black precipitate for c‐fos staining. The second revelation was done using 3,3’‐diaminobenzidine and 0.01% hydrogen peroxide which led to a brown precipitate for TH staining. The reaction was subsequently stopped by rising sections four times with PBS (10 min each). Sections were then mounted on gelatinized slides, dried and dehydrated in increasing gradients of ethanol, cleared in toluene, and were cover‐slipped with Depex.

*Antibodies*: For primary antibodies, rabbit anti‐TH (1:1000, Sigma‐Aldrich, USA), rabbit anti‐c‐fos (1:10 000, Sigma‐Aldrich, USA) and rabbit anti‐GFP (1:2000, Sigma‐Aldrich, USA) were used. For secondary antibodies, anti‐rabbit biotinylated antibody (1:1000, Jackson ImmunoResearch laboratories) was used.

### Statistical Analysis

All data were represented in the text body as mean ± SEM. Sample sizes were determined to be comparable to several previous studies that used optogenetics and/or chemogenetics to study neural mechanism of sleep/wake regulation.^[^ ^27^, ^29^ ^]^ All statistical analyses were performed with in‐built functions of excels, the statistics and Machine Learning toolbox in MATLAB (Mathworks), or Sigma Stat. *n*’s, *p* values, and the kind of test used were provided in the figure caption. Paired and unpaired *t* tests were used for single value comparisons. One‐way analysis of variance (ANOVA) was used to compare more than two groups. Two‐way repeated measures ANOVA was used to perform group comparisons with multiple measurements. Regression analysis and Spearman test were used for correlations. Data were considered to be statistically significant if *p* < 0.05. Bonferroni post hoc correction was used to control for multiple comparisons where appropriate. Figures were prepared using Prism 6.01.

## Conflict of Interest

The authors declare no conflict of interest.

## Author Contributions

K.F. and M.Y. conceived and designed the project. K.F. performed all experiments. K.F. and A.E.F. performed SD experiments. A.E.F. participated with K.F. to perform immunostainings. Y.C. prepared viral constructs. K.F. analyzed data and made the figures. K.F. wrote the manuscript with significant inputs from Y.M. and Y.C. All authors read and approved the final manuscript. M.Y. supervised all aspects of the project.

## Supporting information

Supplemental Information

## Data Availability

The data that support the findings of this study are available from the corresponding author upon reasonable request.
