# Supplementary material for: Motivational and Valence‐Related Modulation of Sleep/Wake Behavior are Mediated by Midbrain Dopamine and Uncoupled from the Homeostatic and Circadian Processes
Source: Adv Sci (Weinh). 2022 Jul 6;9(24):2200640. doi: 10.1002/advs.202200640 (PMC9403635; doi:10.1002/advs.202200640)
Supplement: Supplementary file 1 — Supplemental Information [file ADVS-9-2200640-s001.pdf]

ADVANCED SCIENCE
Open Access

Supporting Information

for *Adv. Sci.*, DOI 10.1002/advs.202200640

Motivational and Valence-Related Modulation of Sleep/Wake Behavior are Mediated by Midbrain Dopamine and Uncoupled from the Homeostatic and Circadian Processes

Karim Fifel, Amina El Farissi, Yoan Cherasse and Masashi Yanagisawa**

Supporting Information

Motivational and Valence-related Modulation of Sleep/Wake Behavior are Mediated by Midbrain Dopamine and Uncoupled from the Homeostatic and Circadian Processes.

Karim Fifel[1]*, Amina El Farissi[1], Yoan Cherasse[1], Masashi Yanagisawa[1]*

1. International Institute for Integrative Sleep Medicine (WPI-IIIS), University of Tsukuba, Tsukuba, Japan

*Correspondence: fifel-k@hotmail.com or yanagisawa.masa.fu@u.tsukuba.ac.jp.

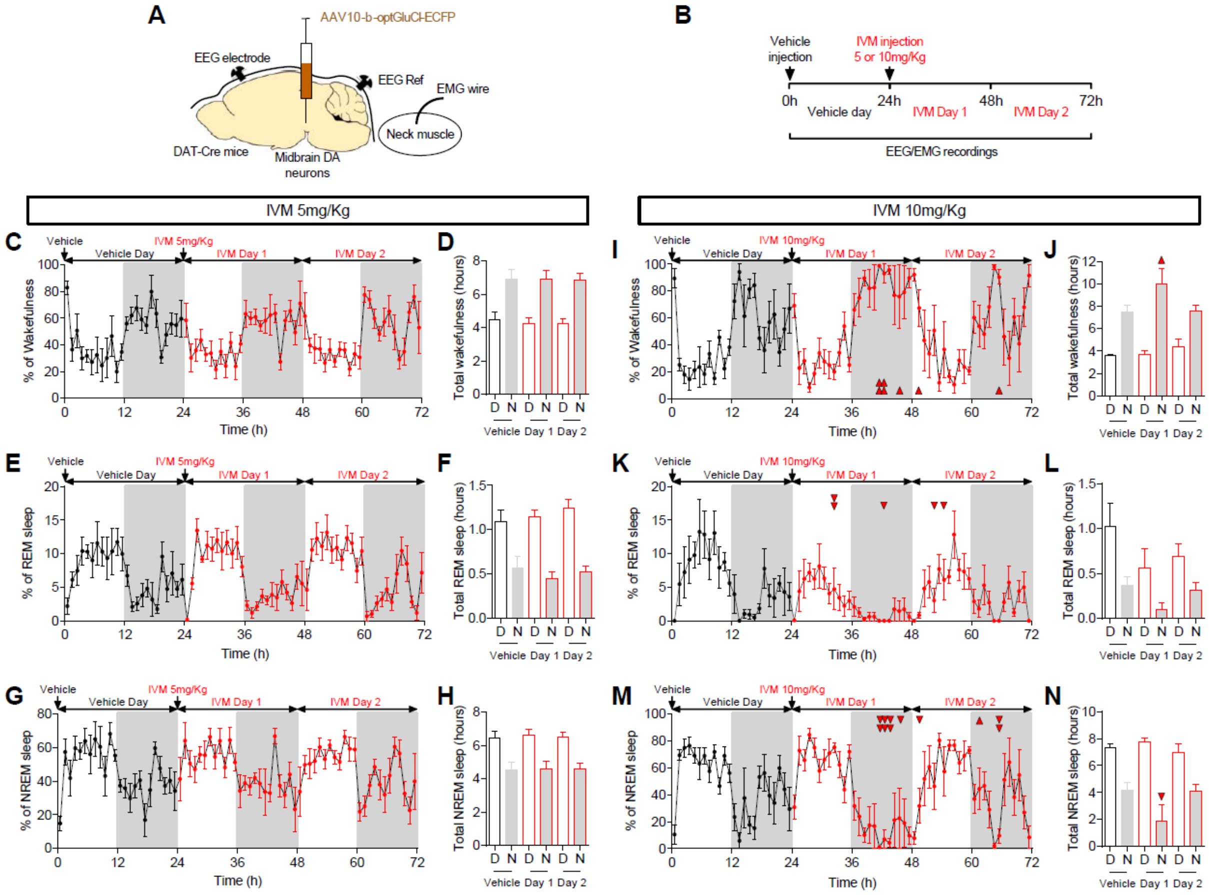

Figure S1. Control experiments for IVM dose in VTA$^{GluCl\beta}$ mice. Related to all main figures.

(**A**) DAT-Cre mice were injected with AAV10-β-optGluCl-ECFP alone (β subunit) into VTA and implanted with EEG and EMG electrodes to record sleep/wake states.

(**B**) Diagram depicting the protocol of testing the impact of 5 and 10mg/Kg of IVM on sleep/wake cycle in VTA$^{GluCl\beta}$ sham mice (n=4).

(**C-H**) Percentage of time spent in wakefulness (**C**), REM (**E**) and NREM sleep (**G**) 1 day before, and 2 days following 5mg/Kg IVM treatment of VTA$^{GluCl\beta}$ sham mice (n=4, Two-way RM ANOVA revealed no significant days x time interaction, $F_{2,23}$=1.027, *p*=0.436). Unlike in VTA$^{GluCl\alpha\beta}$ mice, 5mg/Kg IVM did not significantly change total time spent in wake (**D**), REM (**F**) and NREM sleep (**H**) in VTA$^{GluCl\beta}$ mice (n=5, Two-way RM ANOVA, $F_{1,2}$(Wake)=0.576, *p*=0.584; $F_{1,2}$(REM)=5.954, *p*=0.473; $F_{1,2}$(NREM)= 0.111, *p*=0.897).

(**I-N**) Same as in (**C-H**) but for the higher 10mg/Kg dose of IVM (n=4, Two-way RM ANOVA revealed significant days x time interactions, $F_{2,23}$(Wake)=2.057, *p*<0.001; $F_{2,23}$(REM)=1.323, *p*=0.11; $F_{2,23}$(NREM)=2.178, *p*<0.001; Bonferroni post hoc analysis, one triangle, *p*<0.05, Two triangles, *p*<0.01). Total time spent in wake (**J**) and NREM sleep (**N**) but not REM sleep (**L**) was respectively increased and decreased during the first night after IVM injection (n=4; Two-way RM ANOVA; $F_{1,2}$(Wake)= 6.627, *p*=0.03; $F_{1,2}$(REM)= 1.895, *p*= 0.23; $F_{1,2}$(NREM)= 6.605, *p*= 0.03, Bonferroni post hoc analysis, one triangle, *p*<0.05).

Data represent mean ± SEM.

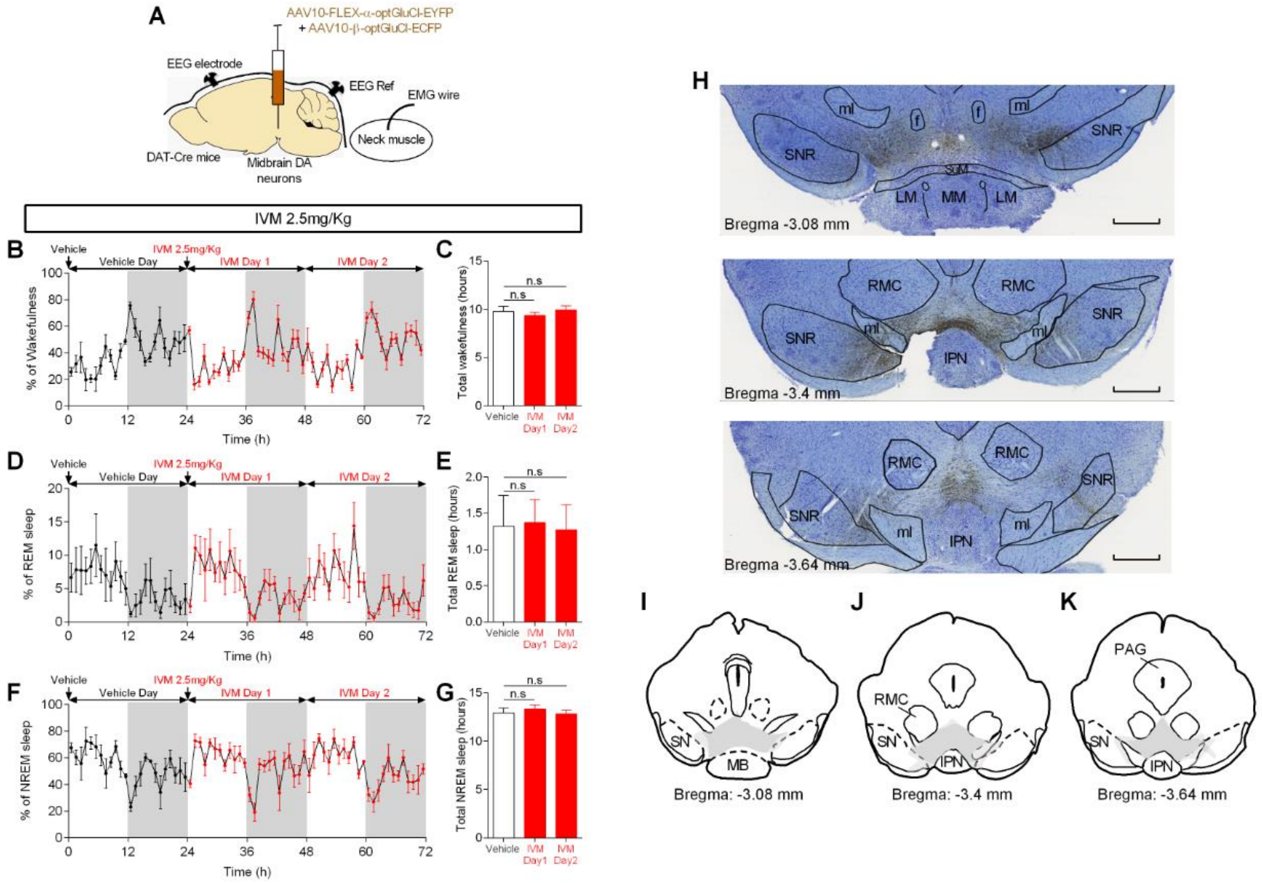

Figure S2. 2.5mg/Kg of IVM does not alter sleep/wake cycle in VTA$^{GluCl\alpha\beta}$ mice. Related to all main figures.

(**A**) DAT-Cre mice were injected with AAV10-FLEX-α-optGluCl-EYFP and AAV10-β-optGluCl-ECFP into midbrain VTA and implanted with EEG/EMG electrodes to follow sleep/wake vigilance states.

(**B-G**) Percentage of time spent in wakefulness (**B**), REM (**D**), NREM sleep (**F**) 1 day before, and 2 days after 2.5mg/Kg IVM treatment of VTA$^{GluCl\alpha\beta}$ mice (n=6, Two-way RM ANOVA revealed no significant days x time interaction, $F_{2,23}$(Wake)=1.386, *p*=0.068; $F_{2,23}$(REM)=1.124, *p*=0.291; $F_{2,23}$(NREM)=1.383, *p*=0.07). Total time spent in wake (**C**), REM (**E**) and NREM sleep (**G**) per day was not significantly different (n=5, One-way RM ANOVA, $F_{2,8}$(Wake)=2.709, *p*=0.126; $F_{2,8}$(REM)=0.452, *p*=0.652; $F_{2,8}$(NREM)=3.689, *p*=0.073).

(**H**) Anti-GFP staining revealing the extent of virus infection in the midbrain. Background counterstaining is Cresyl Violet. Scale bar: 500 µm.

(**I-K**) Drawings of coronal brain-atlas diagrams showing superimposed AAV-infected areas of seven VTA$^{GluCl\alpha\beta}$ mice at Bregma -3.08mm (**I**), -3.4mm (**J**) and -3.64mm (**K**).

Data represent mean ± SEM. ml, medial lemniscus; f, fornix; SNR, Substantia Nigra Reticulata; SN, Substantia Nigra; SuM, Supramammillary nucleus; MM, Medial mammillary nucleus; LM, Lateral mammillary nucleus; IPN, Interpeduncular nucleus; RMC, Red magnocellular nucleus; PAG, Periaqueductal gray.

Data represent mean ± SEM.

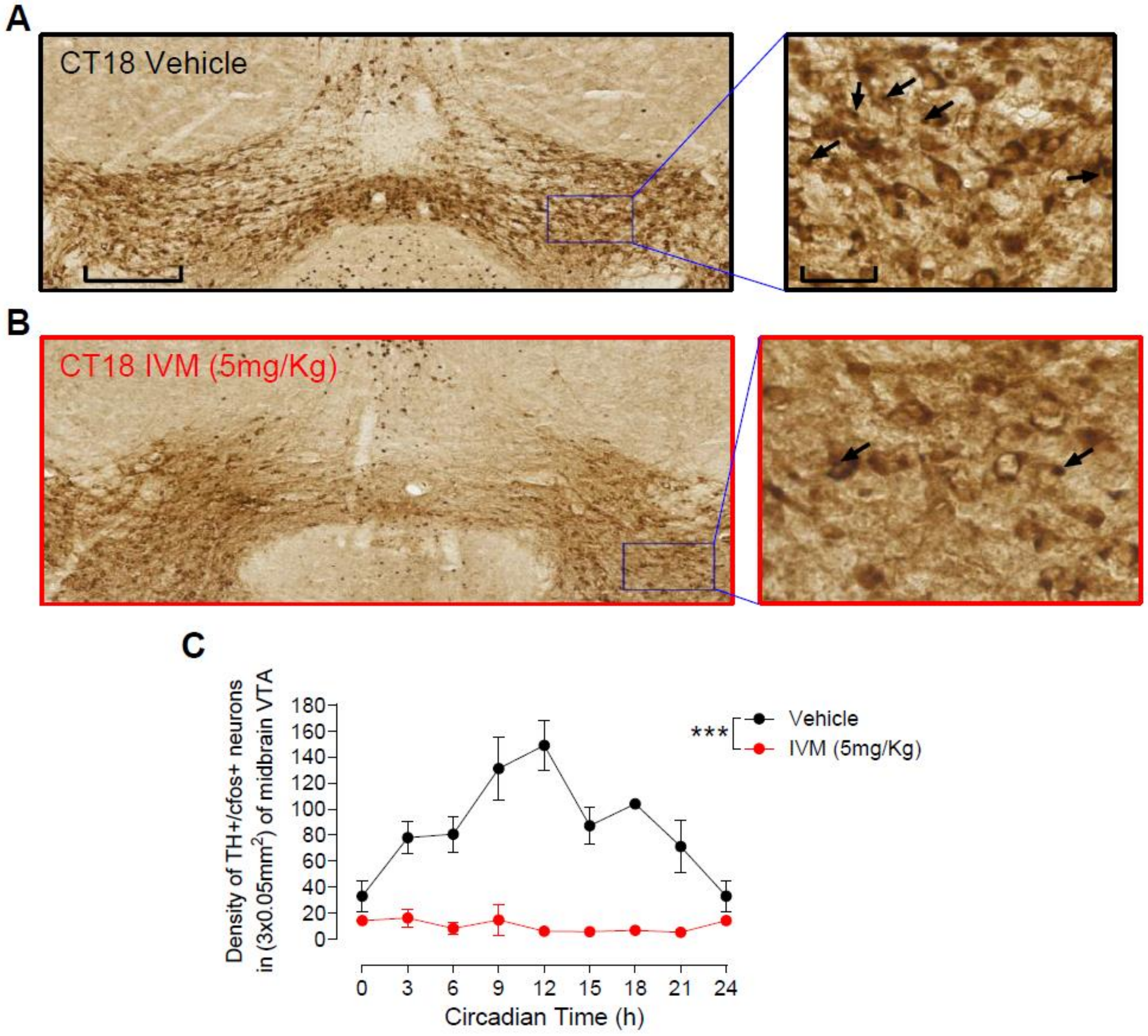

Figure S3. The GluCl/IVM system is efficient in silencing midbrain DA neurons. Related to all main figures.

(**A**,**B**) Representative photographs of the double c-fos/TH immunostaining in the midbrain of vehicle-treated (**A**) and IVM-treated $VTA^{GluCl\alpha\beta}$ mice (**B**) at CT18 of the second day after vehicle or IVM injection. Higher magnification images are shown on the right of each figure (arrows point to c-fos staining). Scale bar on the left: 250µm, on the right: 50µm.

(**C**) Immunohistological quantifications showed that IVM efficiently silenced midbrain DA neurons as revealed by the significant reduction of the number of c-fos+/TH+ neurons throughout the entire second day following IVM/vehicle injections (n=3 per time point, Two-way ANOVA revealed significant days x time interaction, $F_{1,7}$= 5.061, ****p*<0.001).

Data represent mean ± SEM.

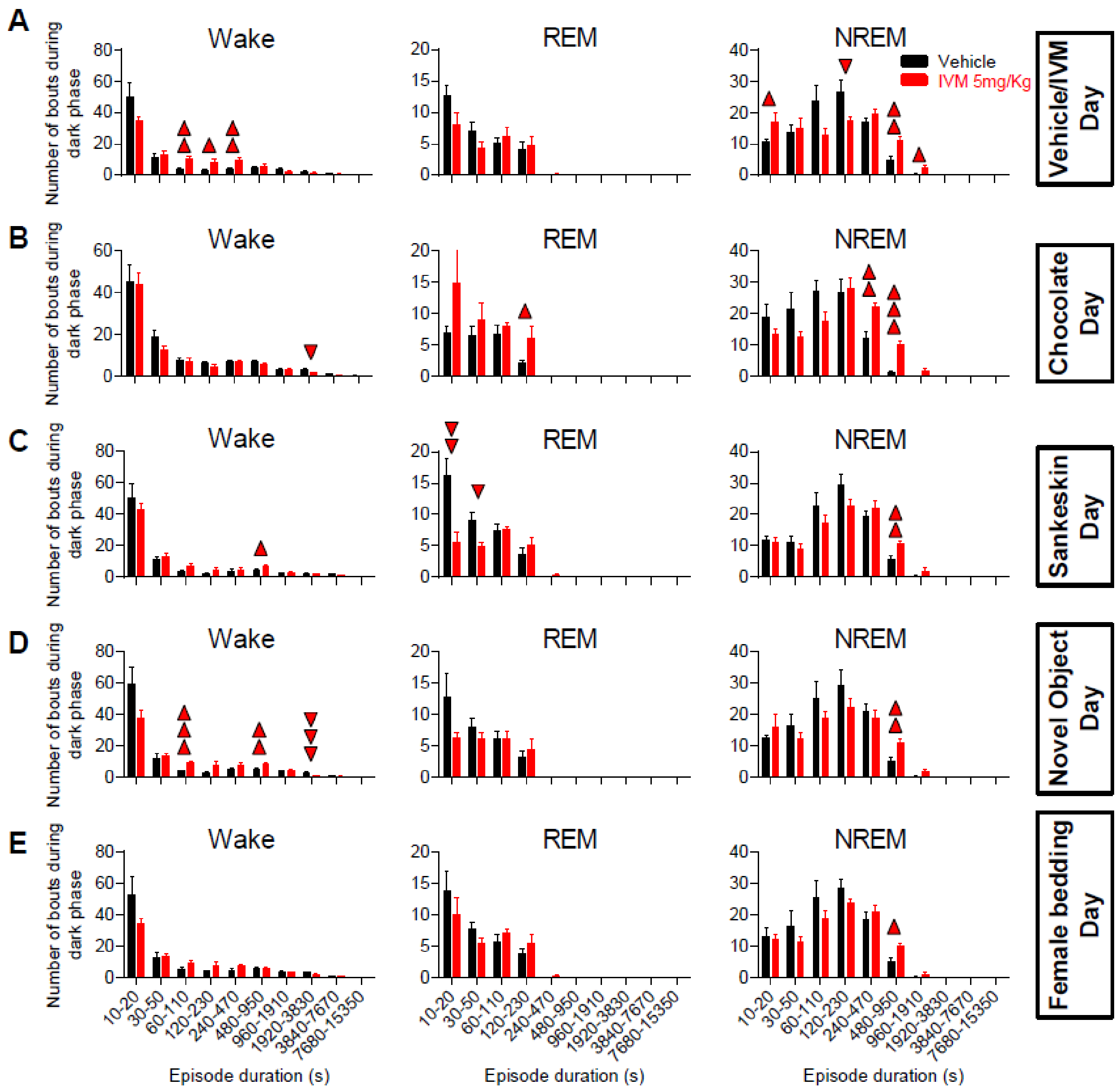

Figure S4. Sleep/wake architecture in vehicle and IVM-treated VTA$^{GluCl\alpha\beta}$ mice during exposure to different salient stimuli. Related to **Figure 1**.

(**A**) Number of wake (left), REM (middle) and NREM sleep (right) bouts with different duration during the dark phase of the baseline day without exposure to any salient stimulus (n=8 for vehicle and 6 for IVM-treated mice, ANOVA test, 1 triangle p<0.05; 2 triangles, p<0.01; 3 triangles p<0.001).

(**B**) Same as **A** for chocolate-exposure day.

(**C**) Same as **A** for snakeskin shed-exposure day.

(**D**) Same as **A** for novel object-exposure day.

(**E**) Same as **A** for female bedding-exposure day.

Data represent mean ± SEM.

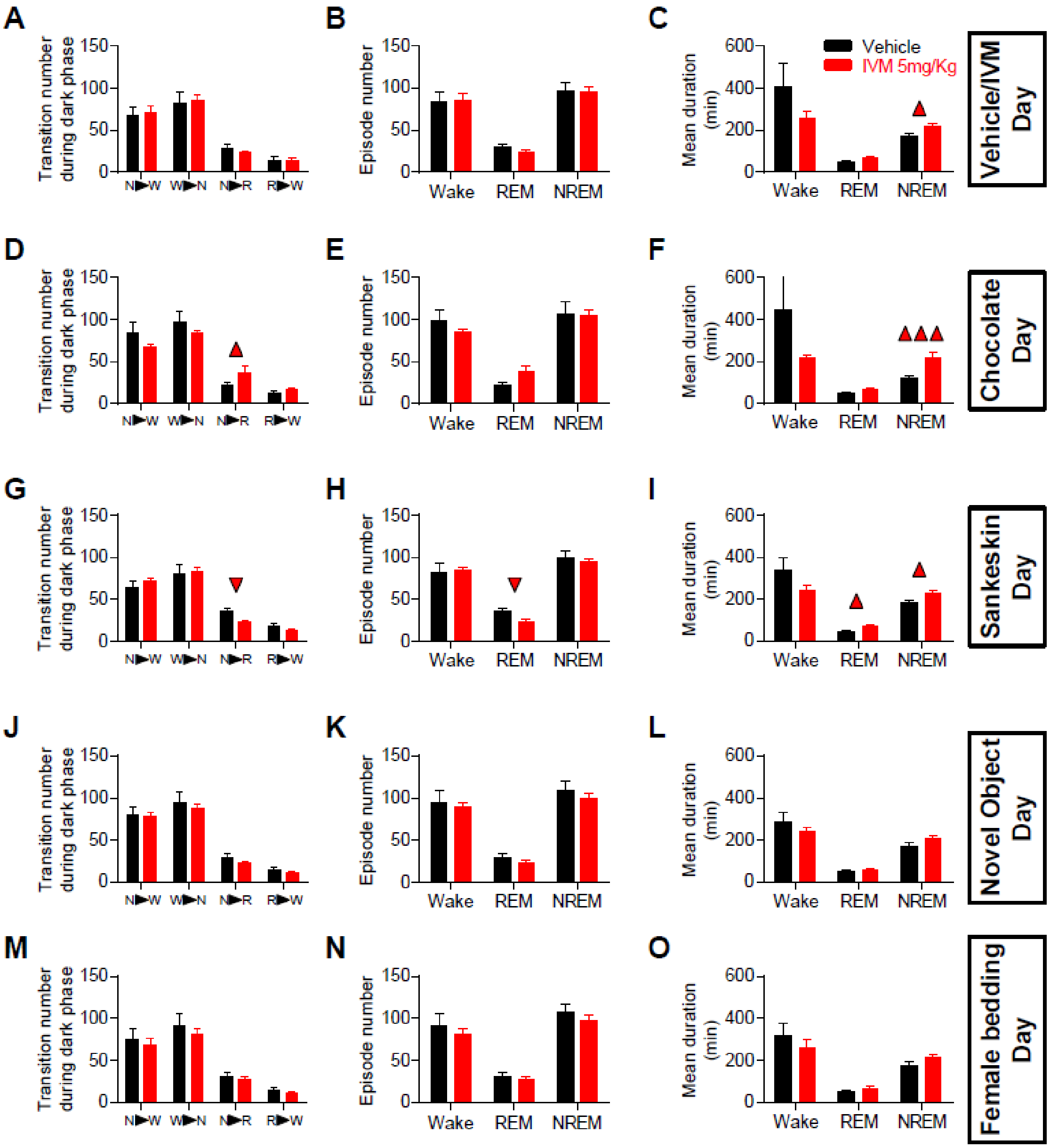

Figure S5. Sleep/wake architecture in vehicle and IVM-treated VTA$^{GluCl\alpha\beta}$ mice during exposure to different salient stimuli. Related to **Figure 1**.

(**A-C**) Number of transitions between different vigilance states (**A**), episode numbers of sleep/wake states (**B**) and mean duration of sleep/wake states (**C**) during the dark phase of the baseline day with no exposure of any salient stimulus (vehicle, n=8; IVM, n=6; ANOVA test, 1 triangle *p*<0.05; 2 triangles, *p*<0.01; 3 triangles *p*<0.001).

(**D-F**) Same as (**A-C**) but for chocolate-exposure day.

(**G-I**) Same as (**A-C**) but for snakeskin shed-exposure day.

(**J-L**) Same as (**A-C**) but for novel object-exposure day.

(**M-O**) Same as (**A-C**) but for female bedding-exposure day.

Data represent mean ± SEM.

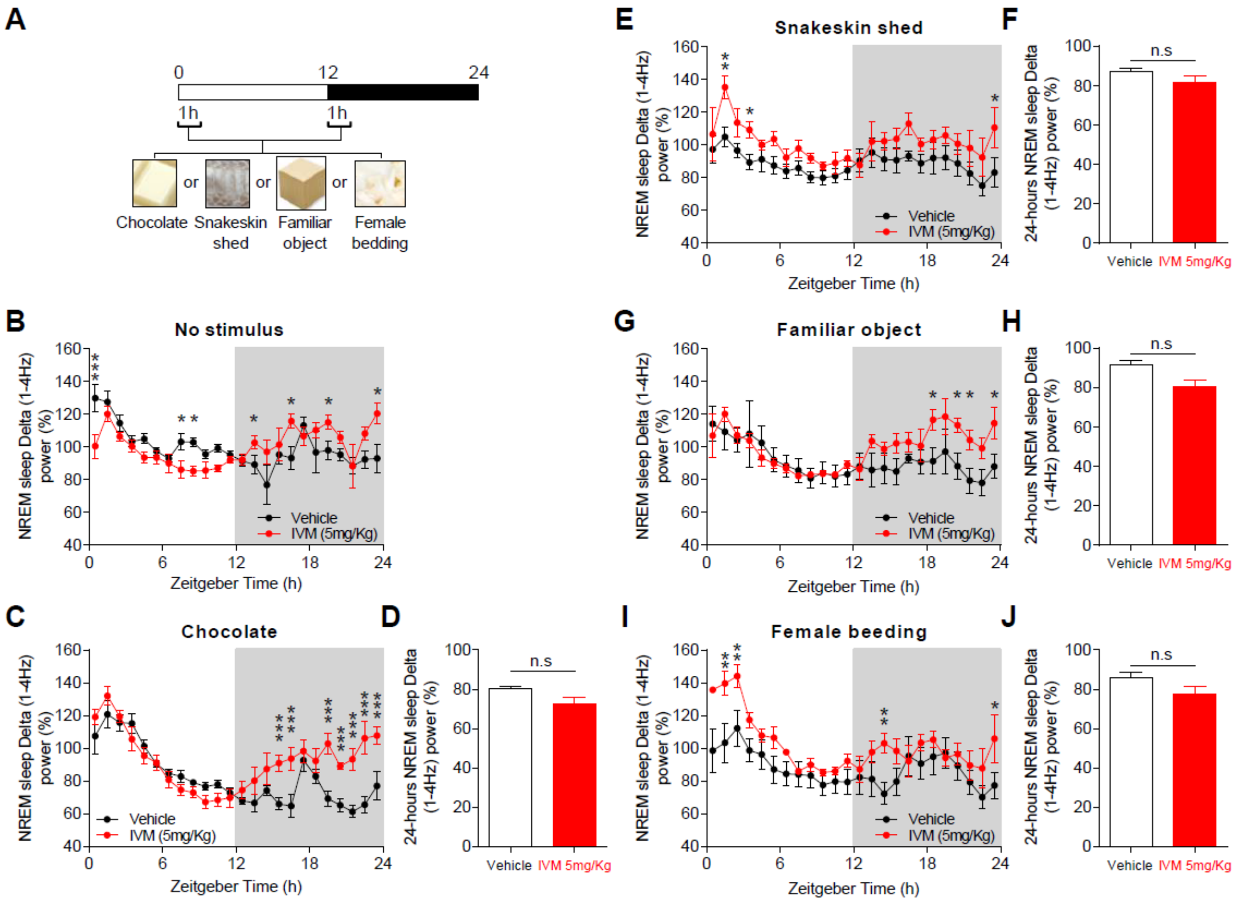

Figure S6. Dynamic of slow wave activity (SWA) under different motivational contexts. Related to **Figure 2**.

(**A**) Diagram depicting experimental protocol. Same as in **Figure 1F**.

(**B**) Hourly evolution of NREM sleep delta power during baseline (no stimulus) day.

(**C, D**) Hourly evolution (**C**) of, and overall 24hrs (**D**) NREM sleep delta power during chocolate-exposure day.

(**E, F**) Same as (**C**, **D**) but for snakeskin shed-exposure day.

(**G, H**) Same as (**C**, **D**) but for novel object-exposure day.

(**I, J**) Same as (**C**, **D**) but for female bedding-exposure day.

(**B**, **C**, **E**, **G**, **I**, Two-way RM ANOVA revealed significant Groups x time interactions. $F_{1,23}$(No stimulus)=2.771, *p*<0.001; $F_{1,23}$(chocolate)=4.214, *p*<0.001; $F_{1,23}$(snakeskin shed)=0.459, *p*=0.986; $F_{1,23}$(familiar object)=0.987, *p*=0.483; $F_{1,23}$(female bedding)=0.792, *p*=0.74. Bonferroni post hoc analysis, **p*<0.05, ***p*<0.01, ****p*<0.001; For **D**, **F**, **H** and **J**; unpaired *t* test).

Data represent mean ± SEM.

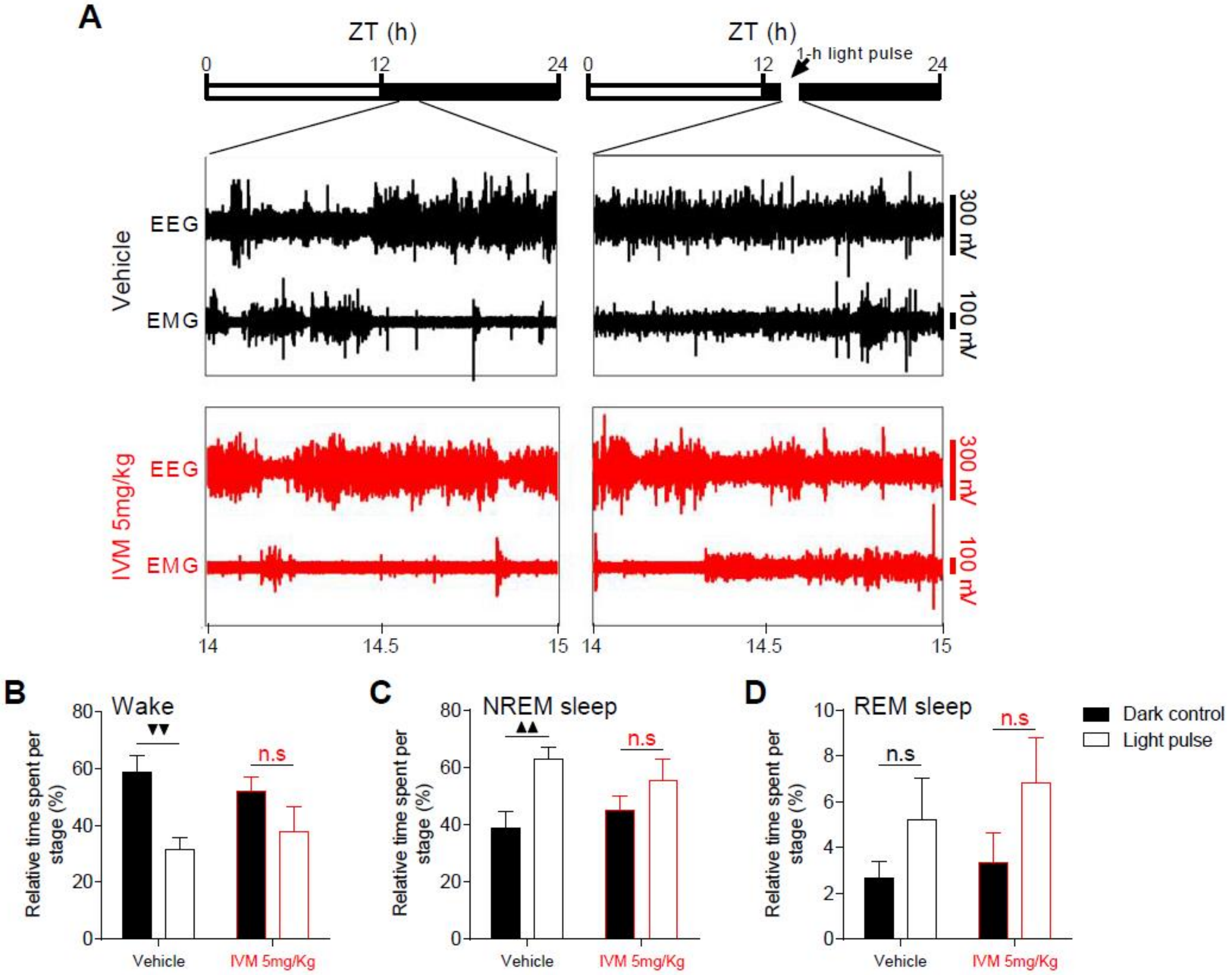

Figure S7. Inhibition of mDA neurons impairs the masking effect of light at ZT14-15. Related to **Figure 4**.

(**A**) Representative EEG and EMG traces of vehicle- (upper) and IVM-treated (lower) VTA$^{GluCl\alpha\beta}$ mice at ZT14-15 without (left) and with (right) light pulse exposure. Light schedules are depicted by black and white bars above the panels.

(**B-D**) Quantification of photic effects on different vigilance states at ZT14-15 (vehicle, n=8; IVM, n=6, Two-way ANOVA, $F_{1,1}$(wake)=1.08, p=0.31; $F_{1,1}$(NREM)=1.41, p=0.25; $F_{1,1}$(REM)=0.09, p=0.772; Bonferroni post hoc analysis, 2 triangles, p<0.01).

Data represent mean ± SEM.

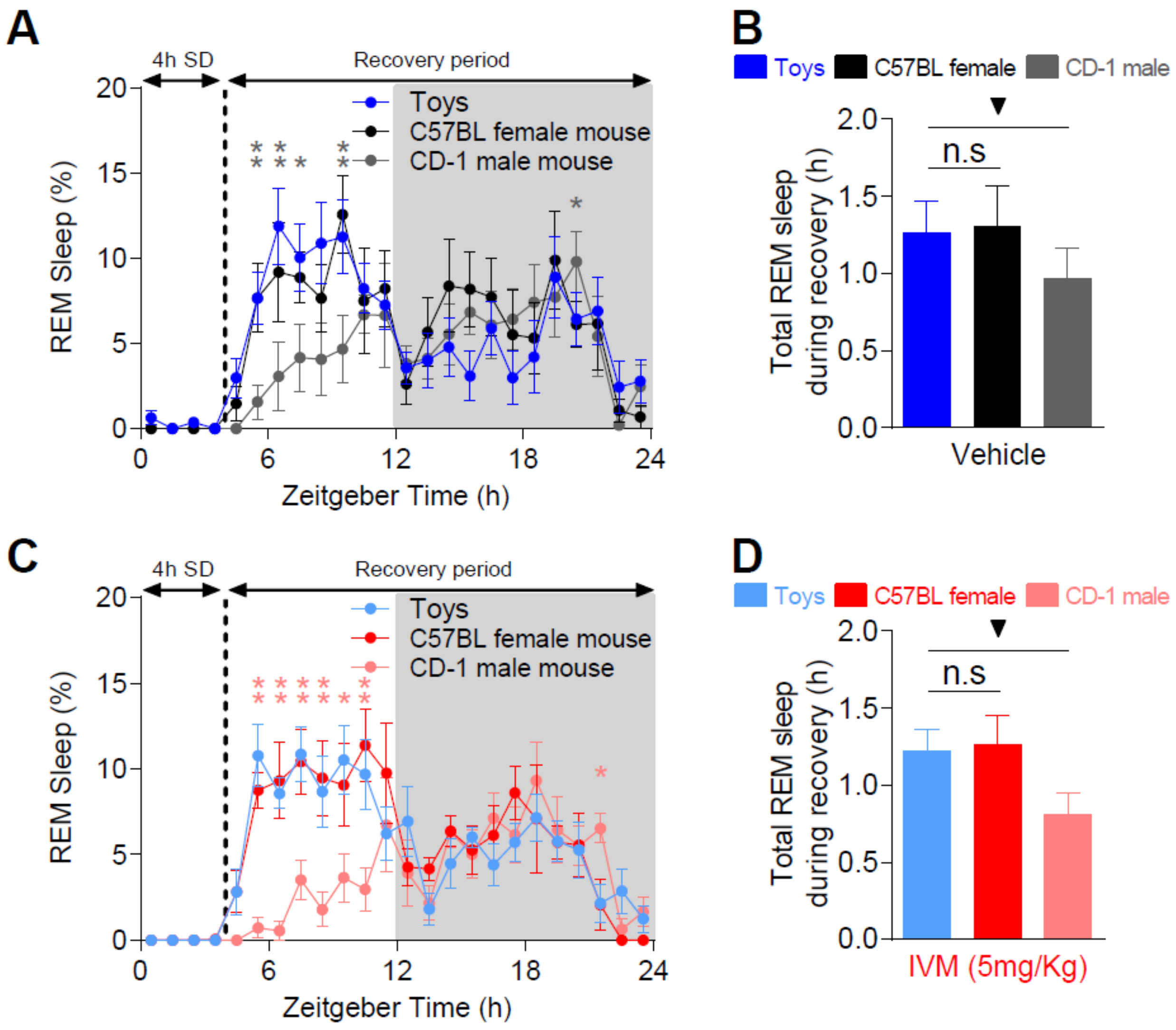

Figure S8. mDA neuron inhibition does not affect valence-related modulation of REM sleep. Related to **Figure 6**.

(**A**, **C**) Percentage of time spent in REM sleep in vehicle (**A**) and IVM-treated (**C**) VTA$^{GluCl\alpha\beta}$ mice during and after SD with Toys, CD1-male or conspecific female interaction (n=6 per group, Two way RM ANOVA revealed a significant Group x time interaction. $F_{1,23}$(vehicle)=2.397, p<0.001, $F_{1,23}$(IVM)=4.11, p<0.001, Bonferroni post hoc analysis, *p<0.01, **p<0.001).

(**B**, **D**) Total time spent in REM sleep in vehicle (**B**) and IVM-treated (**D**) VTA$^{GluCl\alpha\beta}$ mice during recovery period (One-way RM ANOVA, $F_{2,10}$(vehicle)=5.940, $F_{2,10}$(IVM)=2.983. Bonferroni post hoc analysis,▼p<0.05).

Data represent mean ± SEM.

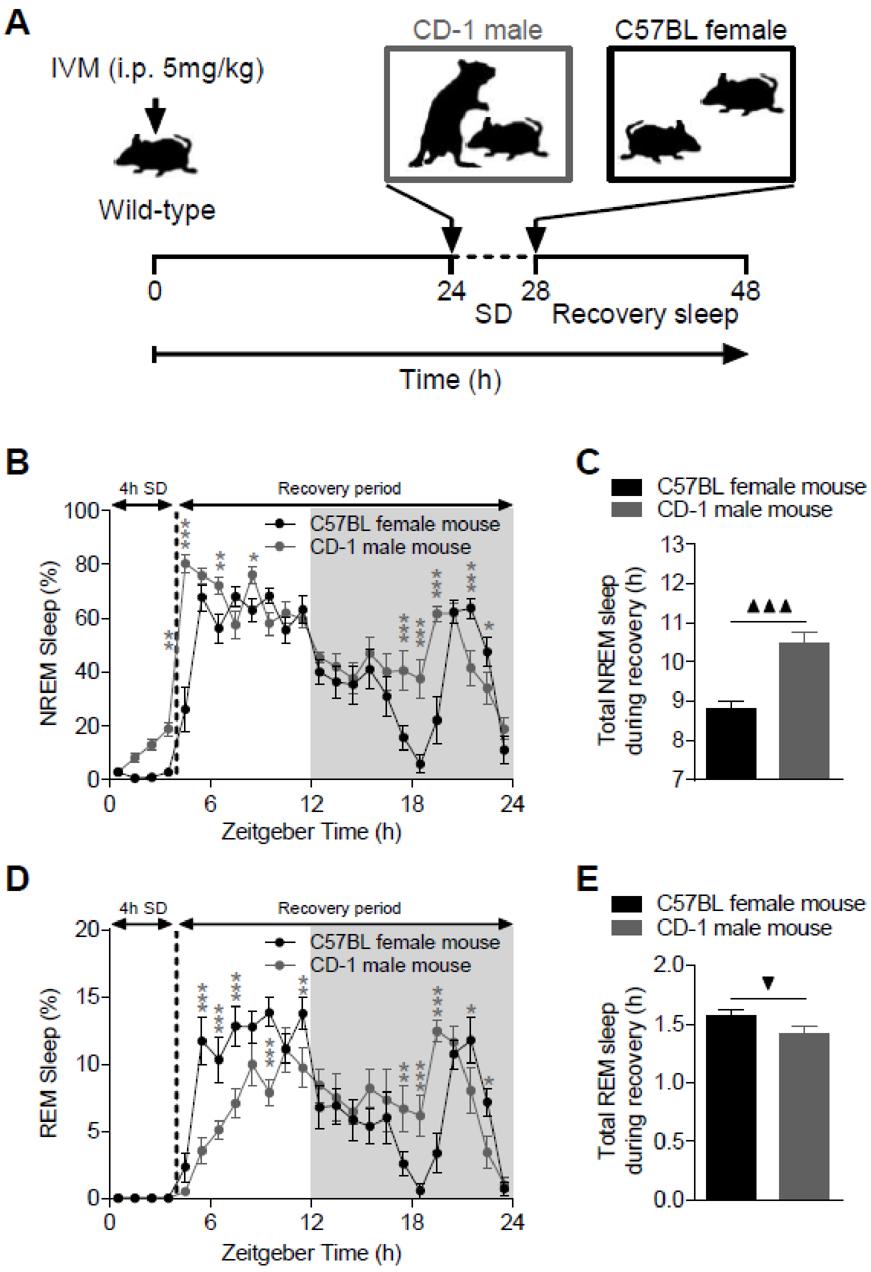

Figure S9. IVM treatment does not affect valence-related modulation of sleep/wake behavior in wild-type mice. Related to **Figure 6**.

(**A**) 24hours following IVM (i.p. 5mg/kg) treatment, wild-type mice were subjected to 4hSD through the interaction with either CD-1 male or a virgin C57BL female.

(**B**) Percentage of time spent in NREM sleep in IVM-treated wild-type mice during and after SD with CD-1 male or conspecific female interaction (n=11, Two-way RM ANOVA revealed significant Treatment x Time interaction ($F_{1,23}$=7.368, p<0.001), Bonferroni post hoc analysis, *p<0.05, **p<0.01, ***p<0.001).

(**C**) Overall amount of NREM sleep during recovery period after SD with CD-1 male and conspecific female interaction in wild-type mice (One-way RM ANOVA, $F_{1,10}=48.031$, $p<0.001$, Bonferroni post hoc analysis, ▲▲▲$p<0.001$).

(**D**) Percentage of time spent in REM sleep in IVM-treated wild-type mice during and after SD with CD-1 male or conspecific female interaction (n=11, Two-way RM ANOVA revealed significant Treatment x Time interaction ($F_{1,23}=6.569$, $p<0.001$), Bonferroni post hoc analysis, *$p<0.05$, **$p<0.01$, ***$p<0.001$).

(**E**) Overall amount of REM sleep during recovery period after SD with CD-1 male and conspecific female interaction in wild-type mice (One-way RM ANOVA, $F_{1,10}=5.925$, $p=0.035$, Bonferroni post hoc analysis, ▼$p<0.05$).

Data represent mean ± SEM.

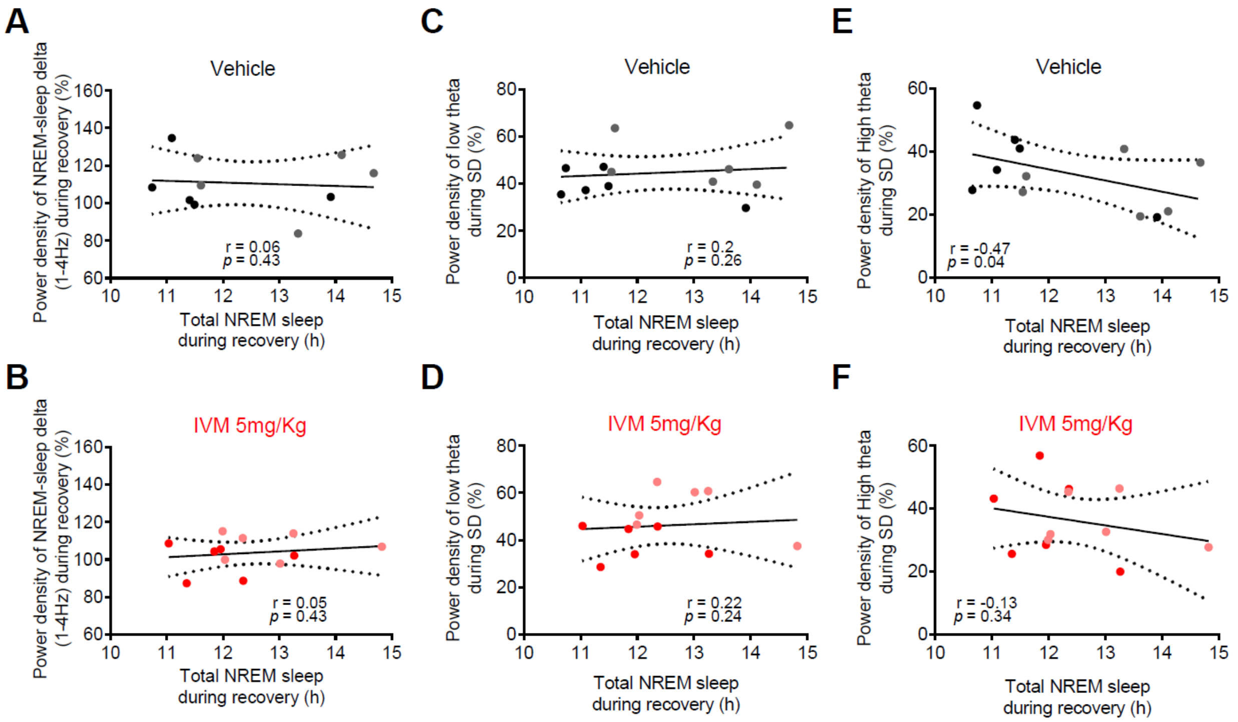

Figure S10. Correlations between EEG density and NREM sleep during recovery period. Related to **Figure 7**.

(**A**, **B**) No significant correlation was found between delta power and NREM sleep amount during recovery period after SD in both vehicle (**A**) and IVM-treated VTA$^{GluCl\alpha\beta}$ mice (**B**) (Spearman correlation).

(**C**, **D**) The power density of low theta (4-8Hz) during SD correlated positively with the total NREM sleep during recovery in vehicle- (**E**) but not after IVM treatment of VTA$^{GluCl\alpha\beta}$ mice (**D**) (Spearman correlation).

(**E**, **F**) The power density of high theta (8-12Hz) during SD correlated negatively with the total NREM sleep during recovery in vehicle (**E**) and IVM-treated VTA$^{GluCl\alpha\beta}$ mice (**F**) (Spearman correlation).
